# CD46 facilitates entry and dissemination of human cytomegalovirus

**DOI:** 10.1038/s41467-019-10587-1

**Published:** 2019-06-20

**Authors:** Kathryn R. Stein, Thomas J. Gardner, Rosmel E. Hernandez, Thomas A. Kraus, James A. Duty, Iban Ubarretxena-Belandia, Thomas M. Moran, Domenico Tortorella

**Affiliations:** 1 Department of Microbiology, Icahn School of Medicine at Mount Sinai, New York, NY 10029 USA; 2 Center of Therapeutic Antibody Development, Icahn School of Medicine at Mount Sinai, New York, NY 10029 USA; 3 Department of Pharmacological Sciences, Icahn School of Medicine at Mount Sinai, New York, NY 10029 USA; 4 Instituto Biofisika (UPV/EHU, CSIC), University of the Basque Country, Leioa, E-48940 Spain; 5 Ikerbasque, Basque Foundation for Science, Bilbao, 48013 Spain

**Keywords:** Herpes virus, Viral infection

## Abstract

Human cytomegalovirus (CMV) causes a wide array of disease to diverse populations of immune-compromised individuals. Thus, a more comprehensive understanding of how CMV enters numerous host cell types is necessary to further delineate the complex nature of CMV pathogenesis and to develop targeted therapeutics. To that end, we establish a vaccination strategy utilizing membrane vesicles derived from epithelial cells to generate a library of monoclonal antibodies (mAbs) targeting cell surface proteins in their native conformation. A high-throughput inhibition assay is employed to screen these antibodies for their ability to limit infection, and mAbs targeting CD46 are identified. In addition, a significant reduction of viral proliferation in CD46-KO epithelial cells confirms a role for CD46 function in viral dissemination. Further, we demonstrate a CD46-dependent entry pathway of virus infection in trophoblasts, but not in fibroblasts, highlighting the complexity of CMV entry and identifying CD46 as an entry factor in congenital infection.

## Abstract (web summary)

Human cytomegalovirus (CMV) infects a wide range of host cells. Here, using a high throughput antibody screening platform, the authors identify the cell surface receptor CD46 to be required for CMV infection of epithelial cells and trophoblast-derived cells, the latter critical for congenital CMV infection.

## Introduction

Human cytomegalovirus (CMV) is implicated in diseases ranging from atherosclerosis, rheumatoid arthritis, and Alzheimer’s disease in the elderly^1^, pneumonia, colitis, retinitis, hepatitis in AIDS and transplant patients,^2^ and is the leading cause of viral birth defects worldwide^3^. During acute CMV disease, essentially any organ can experience symptoms and present histological evidence of active virus infection. Varying degrees of CMV replication efficiency have been observed in epithelial cells, neuronal cells, macrophages, smooth muscle cells, fibroblasts, endothelial cells, hepatocytes, and dendritic cells^4^. Despite its wide tropism, the vast majority of CMV research examining entry has been performed utilizing human fibroblast cell lines. This has provided a strong foundation for our understanding of CMV entry, yet the complexity of CMV infection suggests the existence of numerous uncharacterized entry factors.

CMV infection requires viral and host proteins in a complex mechanism of entry that varies across cell types that provide the virus with a wide tropism. The envelope proteins of the virion engage with cellular proteins that act as binding factors and receptors, and include heparin sulfate proteoglycans (HSPGs)^5^, integrins^6^, epidermal growth factor receptor (EGFR)^7,8^, platelet-derived growth factor receptor (PDGFR)^9^, THY-1 cell surface antigen (CD90)^10^, and recently neuropilin-2 (Nrp2)^11^, CD147^12^ and OR14I1^13^. Envelope proteins gB and gM/N initially tether to HSPGs. Binding is then described as an interaction between the oligomer gB, trimer complex gH/gL/gO, the pentamer complex gH/gL/UL130/131 (PC) and entry receptors PDGFR, EGFR, integrins, and CD90^14^. It is proposed that entry into fibroblasts then occurs at the cell surface by membrane fusion/macropinocytosis in a pH-independent manner involving gB and the trimer. In contrast, entry into epithelial, endothelial, dendritic, and monocytic cells occurs within the endosome and/or by macropinocytosis in a pH-dependent manner facilitated by gB, the trimer, and the PC^15^. Cell-surface factors that participate in virus infection and cell-to-cell spread are not well defined in a cell-type specific manner, and are essential to understanding basic principles of virus entry and development of effective therapeutics^16^. During CMV entry, the identification of direct interactions between the trimer and PDGFRα and the PC and Nrp2 have recently begun to parse out cell-type pathways^11,17–19^. To further address this knowledge gap, we have developed a high-throughput functional screen to identify CMV biologics that can be employed to detect cellular factors involved in virus infection.

We generated a library of monoclonal antibodies (mAbs) directed against human retinal pigmented epithelial (ARPE-19) cell-surface proteins that were assessed for CMV entry inhibition. Importantly, this expansive repertoire of antibodies was isolated from mice immunized with human cell-derived vesicles (CDV), consisting of diverse membrane proteins that activate the mouse humoral response. The mAb library and a high-throughput inhibition assay were utilized to identify CD46 (membrane cofactor protein or MCP) as a new factor for CMV entry. CD46 is a type-1 membrane glycoprotein expressed on all nucleated cells characterized as a complement regulatory protein and as a receptor for several human pathogens, including the vaccine strain of measles virus on Jurkat and Hela cells, adenovirus (groups B and D) infection of epithelial cells, and HHV-6 infection of T cells^20^. We demonstrate that CD46 impacts viral dissemination in epithelial cells and that CD46-dependent entry can occur in trophoblasts, a cell type critical for congenital CMV infection. Additionally, the identification of CD46 as an entry factor supports the effectiveness of our immunization/screening strategy.

## Results

### CDVs generate mAbs to surface proteins

**Fig. 1:**
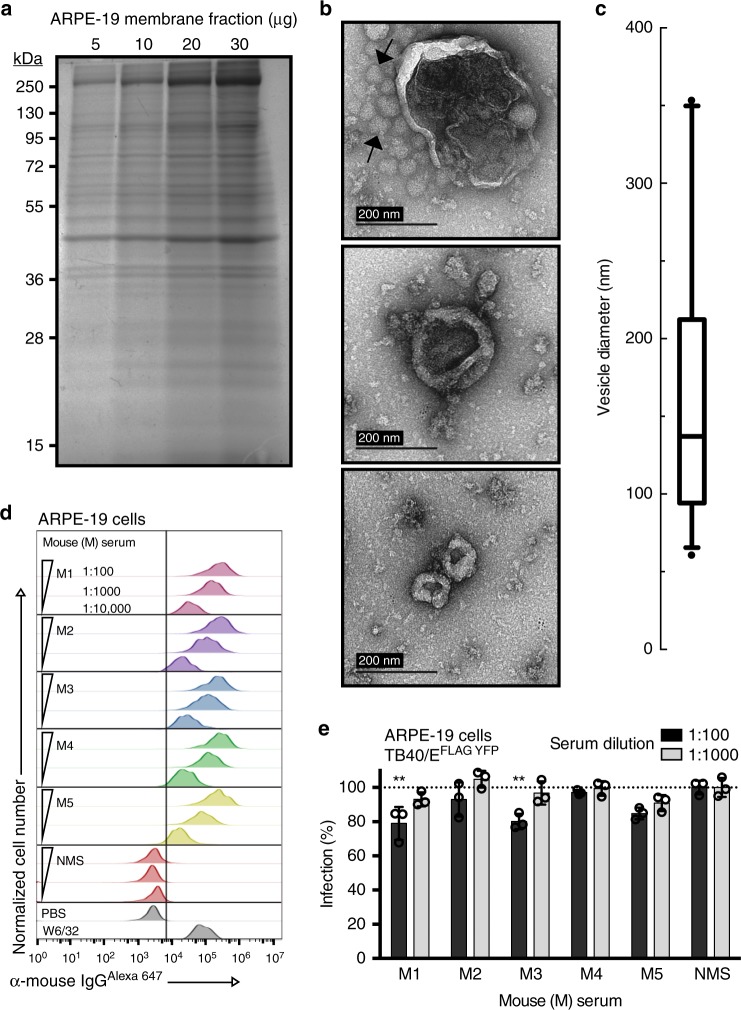
Physical attributes of ARPE-19 cell-derived vesicles and their humoral immune response in mice. **a** Increasing amounts of ARPE-19 membrane fraction (μg) were resolved on a SDS-polyacrylamide gel (12.5%) and visualized using Gel Code Blue Reagent (ThermoFisher Scientific). **b** Transmission electron microscopy (TEM) images of the membrane fraction. Representative images demonstrate the range in vesicle sizes within the fraction. Arrows indicate observed protein aggregates surrounding vesicles. **c** The distribution of vesicle diameters (nm) (*n* = 29). Bounds of the box span 25–75% percentile, the center line represents median, and whickers visualize 5 and 95% of the data points. **d** Dilutions of serum from five mice (M1–M5) immunized with the ARPE-19 membrane fractions were subjected to flow cytometry with ARPE-19 cells. Normal mouse serum (NMS), PBS, and the mAb W6/32 (targeting MHC class I) were used as controls. **e** Two dilutions of serum from the ARPE-19 vesicle immunized mice were subjected to a high-throughput infectivity assay (HTI) with TB40/E^FLAG YFP^ infection of ARPE-19 cells using YFP fluorescence as readout for infection. The % infection was determined using virus incubated with normal mouse serum (NMS) as 100%. Infection data was performed in triplicate. s.d. is depicted in the experiment. **P < 0.01(Student’s two-tailed *t* test)

ARPE-19 cells were selected as the cell target for mAb generation due to entry of CMV requiring the PC and their physiological relevance to CMV-associated diseases^21^. ARPE-19 cell-derived membrane fractions were utilized as an immunogen to elicit a robust humoral response. Analysis of the membrane fraction by SDS-PAGE revealed proteins of varying molecular weights between 10–250 kDa (Fig. 1a). Further assessment with transmission electron microscopy (TEM) revealed that the membrane fraction mostly consisted of CDVs (Fig. 1b). The majority of CDVs being ~100–200 nm (average diameter: 166 nm, SD: 85.7 nm) (Fig. 1c) with ~35% surrounded by protein complex aggregates (Fig. 1b, arrows). Most importantly, we observed an enhanced humoral response when utilizing the membrane fraction, in comparison to whole cells, for immunizing mice (Supplementary Fig. 1a). Thus, we demonstrated that CDVs studded with diverse membrane proteins generate a robust humoral response^22^.

Mice were immunized and boosted with ARPE-19 CDVs. An enhancement of antibody binding for each of these mice against intact ARPE-19 cells (Fig. 1d), in comparison to normal mouse serum (NMS), demonstrated that sera from CDV-immunized mice recognized cell surface proteins. To address whether the sera from the immunized mice can neutralize CMV infection, an inhibition assay was performed with the CMV reporter strain TB40/E^FLAG YFP^ ^23^. While cellular proteins relevant for CMV entry may elicit only a fraction of the humoral response assessed in this assay, the serum from two mice significantly limited virus infection ~20% (Fig. 1e). Mouse #3, possessing an anti-ARPE-19 humoral response and a significant neutralization titre, was selected and the spleen from the animal was utilized to generate 2976 single cell B cell hybridoma clones. Collectively, the combination of a robust humoral response due to CDVs and single cell cloning produced an expansive library of hybridoma clones.

### Classification of mAb library

**Fig. 2:**
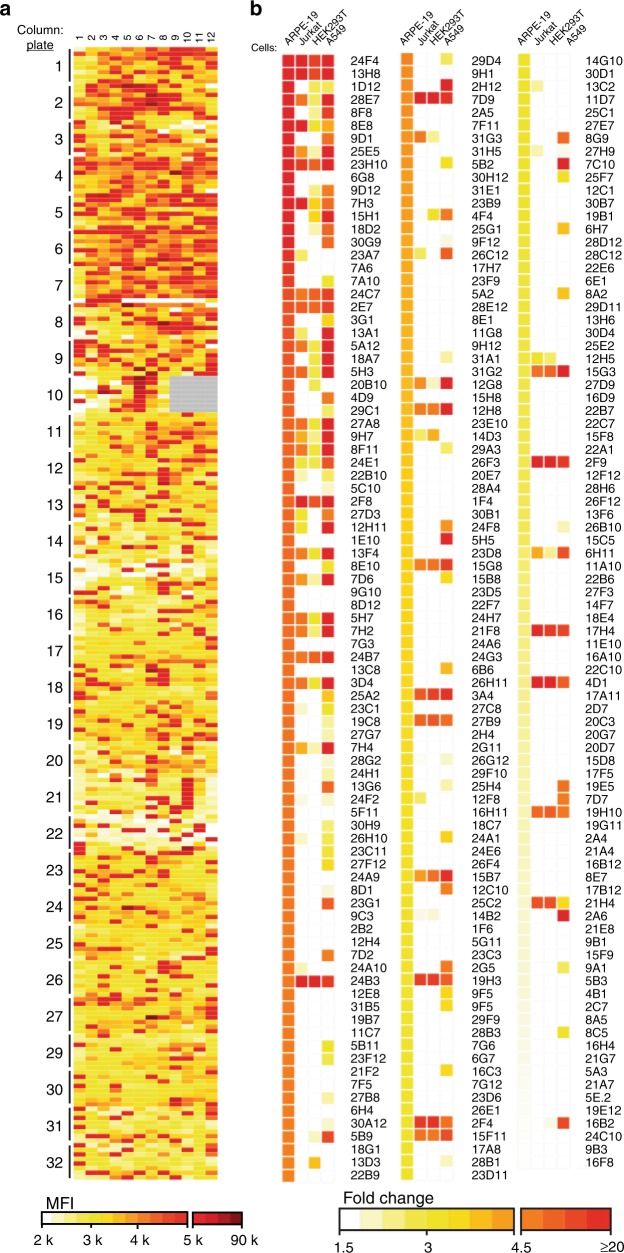
High-throughput screening for cell-surface binding clones. **a** Hybridoma supernatants across 32 96-well plates were incubated with ARPE-19 cells, with binding detected through flow cytometry. Clones that bound with mean fluorescent intensity (MFI) two fold over background (~5 k) or higher were designated as cell-surface binders. Darkening red hues are relative to increasing MFI. Wells without clones are represented in gray. **b** Supernatant from ARPE-19 cell-surface binders was subjected to high-throughput flow cytometry using against Jurkat, HEK293T and A549 cells to evaluate specificity. Fold change of MFI was determined on a cell-type basis compared to a known non-binder (anti-gH 5C3) and is represented by darkening red hues relative to its increase

Supernatant from the hybridoma clones was evaluated for binding to ARPE-19 cells by high-throughput flow cytometry analysis (Fig. 2a). Examination of the fluorescence signal identified 260 clones (~9%) with an enhanced mean fluorescent intensity (MFI) greater than two fold over background, which were categorized as cell-surface binders. Clones that did not bind to the cell surface may target intracellular proteins or recognize linear epitopes. Of the flow cytometry positive clones, the MFI may vary based on expression level of the protein, immunoglobulin concentration in the supernatant, and mAb affinity for surface protein. The analysis intended to exclude non-binding clones and IgM subtypes.

To evaluate diversity among the ARPE-19 cell-surface binding antibody clones, reactivity was examined to other human cell types: T lymphocyte cells (Jurkat), embryonic kidney cells (HEK293T), and alveolar epithelial cells (A549) (Fig. 2b). The affinity profiles for the cell types varied from 52.7% specific for ARPE-19 cells (e.g., 6G8, 7A6, and 1E10) to 15.8% reactive against all cell types (e.g., 24F4, 13H8, and 23H10) (Supplementary Fig. 1b). Interestingly, ~20% of clones bound only to the ARPE-19 and A549 epithelial cell lines. The specificity of these clones to certain cell types highlights the potential of the high-throughput binding assay to identify biomarkers against diverse cells including activated immune cells and cancer cells. Importantly, the diversity of binding profiles of the clones highlights the array of antibodies that target surface proteins.

### CMV inhibiting mAb targets CD46

**Fig. 3:**
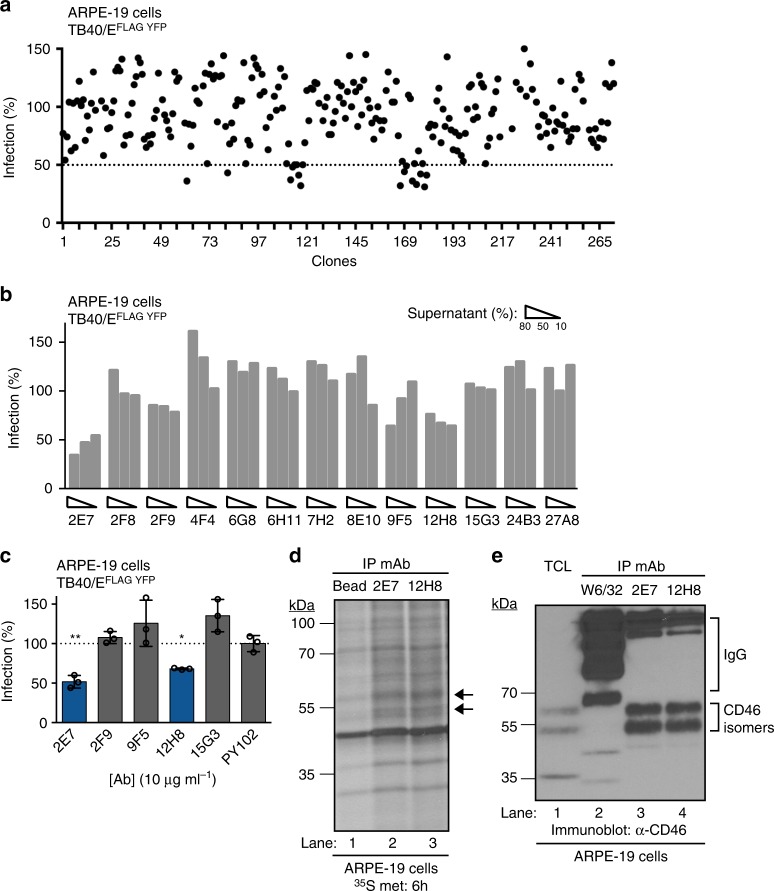
Identification of inhibitory monoclonal antibodies and their cellular target. **a** Supernatants from cell-surface binding clones were subjected to a high-throughput infectivity assay (HTI) with TB40/E^FLAG YFP^ infection of ARPE-19 cells using YFP fluorescence as readout for infection. The % infection was determined using virus incubated with media alone as 100%. **b** Clones demonstrating reduced infection were validated using the TB40/E^FLAG YFP^/ARPE-19 cells HTI with varying amounts (%) of supernatant. **c** Purified mAbs (10 μg ml^−1^) were analyzed using the TB40/E^FLAG YFP^/ARPE-19 HTI in technical triplicates. **d** Polypeptides recovered with mAb 2E7 and 12H8 (arrows) from ARPE-19 cells metabolically labeled with ^35^S-methinionine (6 h) were resolved on a SDS-polyacrylamide gel and visualized on a radiographic film. Beads only was used as a control. **e** Polypeptides recovered with mAb 2E7 and 12H8 from ARPE-19 cells were subjected to immunoblot analysis using anti-CD46 antibodies. mAb W6/32 and total cell lysates (TCL) were included as controls. The polypeptides and molecular weight markers are indicated. s.d. is depicted in the experiment. *P < 0.05, **P < 0.01 (Student’s two-tailed *t* test)

The ARPE-19 cell-surface binding clones were subjected to a high-throughput infectivity assay (HTI) utilizing CMV reporter virus TB40/E^FLAG YFP^. A greater than 50% decrease in virus infection was caused by 25 clones (Fig. 3a), suggesting that these mAbs limit an early step of virus entry. Hybridoma clones were isotyped and thirteen clones were expanded excluding IgM and IgG3 clones and were validated by HTI (Fig. 3b). Clones 2E7, 2F9, 9F5, and 12H8 continued to limit virus infection. Purified immunoglobulin from these clones was evaluated in a TB40/E^FLAG YFP^ mAb inhibition assay (Fig. 3c) and clones 2E7 and 12H8 consistently reduced virus infection.

To identify the protein target of clones 2E7 and 12H8 clones, proteins recovered by immunoprecipitation from ARPE-19 cells metabolically labeled with ^35^S-methionine were resolved by SDS-PAGE. The two observed polypeptides of ~60 and ~50 kDa suggest that both clones bind to identical proteins (Fig. 3d, (lanes 3–4) arrows). In fact, nucleotide sequencing of 2E7 and 12H8 cDNA confirmed that they are identical clones (Supplementary Fig. 1c). Subsequently, two polypeptides corresponding to ~60 and ~50 kDa from a 2E7 immunprecipitation using ARPE-19 cells were excised and subjected to mass spectroscopy identifying isoforms of CD46 (Supplementary Fig. 1d). The CD46 isoforms are consistent with reported alternative splicing events that produce distinct classes of CD46 and result in two prominent polypeptide species (58–68 kDa and 48–56 kDa) dependent on the presence or absence of a STP-rich exon^24^. To further validate the target of clones 2E7 and 12H8, an immunoprecipitation and anti-CD46 immunoblot experiment detected the same two polypeptides compared to total cell lysates (Fig. 3e, compare lane 1 to lanes 3–4). The independent identification of two identical mAb clones that inhibit CMV infection supports the sensitivity of the assay. Based on these results, clone 2E7 was utilized for further characterization of CD46’s role during CMV infection.

### Characterization of CD46-dependent entry

**Fig. 4:**
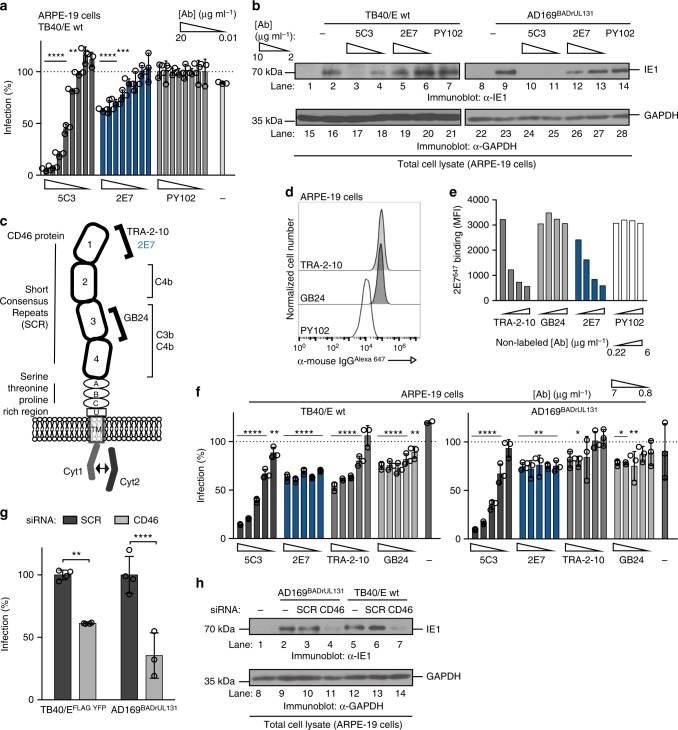
CD46-dependent CMV entry in epithelial cells. **a** TB40/E wt was subjected to a mAb inhibition HTI in ARPE-19 cells with mAb 5C3 (anti-gH), mAb 2E7 (anti-CD46), mAb PY102 (non-binding control) or no-antibody control (20-0.01 μg ml^−1^, in threefold dilutions). Virus infection from PY102-treated cells represents 100% infection. **b** Total cell lysates from uninfected, TB40/E wt and AD169^BADrUL131^ infected ARPE-19 cells treated with mAbs 5C3 or 2E7 (10 and 2 μg ml^−1^), mAb PY102 (10 μg ml^−1^), and no mAb were subject to immunoblot analysis for IE1 and GAPDH. (**c**) CD46 protein model represents four consensus repeats (SCRs) where complement proteins C4b, C3b, and C4b bind, a serine/threonine/proline (STP)-rich region, an uncharacterized segment (U), a transmembrane domain (TM), and a cytoplasmic tail^47^. Alternative splicing accounts for common isomers expressing either STP regions BC or just C and either a short cytoplasmic tail (CYT1) or long cytoplasmic tail (CYT2)^24^. **d** Using flow cytometry, anti-CD46 mAbs TRA-2–10 and GB24 (2 μg ml^−1^) were analyzed on ARPE-19 cells in comparison to PY102. **e** ARPE-19 cells were incubated with labeled 2E7^647^ (2 μg ml^−1^) and increasing concentrations (6.7–0.22 μg ml^−1^, in 3-fold dilutions) of mAbs TRA-2-10 or GB24. The mean fluorescence intensity (MFI) of anti-mouse IgG^Alexa647^ was measured by flow cytometry with PY102 (−) and non-labeled 2E7 (+) as controls. **f** TB40/E wt and AD169^BADrUL131^ infection of ARPE-19 cells treated with mAbs 5C3, 2E7, TRA-2-10, GB24, and PY102 (6.7–0.74 μg ml^−1^, in threefold dilutions) and a no-antibody control were analyzed using an HTI. PY102-treated cells represents 100% infection. **g** ARPE-19 cells transfected with non-targeting siRNA (SCR) or siRNA targeting CD46 (CD46) were infected with TB40/E^FLAG YFP^ and AD169^BADrUL131^ and analyzed for infection (YFP fluorescent intensity) by flow cytometry. SCR-transfected cells represent 100% infection. **h** Cell lysates from ARPE-19 cells transfected with no siRNA, SCR, or CD46 targeting siRNA and infected with AD169^BADrUL131^ or TB40/E wt (MOI:0.5) were subjected to anti-IE1 and GAPDH immunoblots. The polypeptides and molecular weight markers are indicated for the immunoblots. Infection experiments were performed in triplicate. s.d. is depicted in the experiment. *P < 0.05, **P < 0.01, ***P < 0.001, ****P < 0.0001 (Student’s two-tailed *t* test)

Using the CMV infectivity assay, anti-CD46 mAb 2E7 limited virus infection using TB40/E wt in a concentration dependent manner with a maximum inhibition of ~50% (Fig. 4a). In parallel, the anti-CMV gH neutralizing mAb 5C3 almost completely eliminated virus infection. The data indicate that mAb binding to a cellular entry factor in our system, as compared to a viral factor, may not completely inhibit a virus infection based on its role during virus infection as a receptor co-factor or during a latter step of entry. Consistent with this finding, a mAb directed to the CD90 entry factor also only partially inhibited virus infection (Supplementary Fig. 2a). Subsequently, infection with the variant strain AD169^BADrUL131^ (containing a functional UL131 that allows for pentamer expression and the ability to infect epithelial cells^25^) and TB40/E wt was analyzed by western blotting for CMV immediate early-1 (IE1) protein (Fig. 4b, lanes 1–14) and anti-GAPDH was used a loading control (Fig. 4b, lanes 15–28). Aligning with previous data (Fig. 4a), a reduction of IE1 levels to ~50% was observed in 2E7-treated virus-infected cells (Fig. 4b, lanes 5 and 12). As expected, IE1 levels were mostly undetectable in all 5C3 treated infected cells (Fig. 4b, lanes 3–4 and 10–11). These data further demonstrated that anti-CD46 mAb treatment consistently limited virus infection to ~50%. Thus, we propose that CD46 functions as an entry factor that participates in the infection steps such as viral binding, endocytosis, and/or fusion.

Next, previously described anti-CD46 mAbs were utilized to determine the potential CD46 epitope bound by 2E7 and to define the domains important for CD46-dependent CMV entry. Anti-CD46 mouse mAb TRA-2–10^26^ and GB24^27^ are characterized to interact with the SCR1 and SCR3/4 domains of CD46, respectively (Fig. 4c). An initial flow cytometry experiment demonstrated that TRA-2–10 and GB24 bound to cell surface CD46 in ARPE-19 cells (Fig. 4d). In a mAb binding competition experiment, a concentration dependent decrease in 2E7^Alexa647^ MFI was observed in the presence of TRA-2–10, but not for GB24 (Fig. 4e). This suggests that 2E7 and TRA-2–10 compete for a similar domain in the SCR1 region of CD46 (Fig. 4c). Subsequently, these anti-CD46 mAbs were employed to determine their impact on CMV infection. ARPE-19 cells, treated with all three anti-CD46 mAbs, were evaluated for infection with strains TB40/E wt and AD169^BADrUL131^ (Fig. 4f). All three anti-CD46 mAbs significantly reduced virus infection by both CMV strains. Antibody binding was consistently unable to decrease infection greater than 50%, results congruent with previous findings. Overall, these data support that mAb binding to the CD46 extracellular domains SCR1 and SCR3/4 limit CMV infection.

Additionally, we examined whether knocking down CD46 with siRNA impacted virus infection. The loss of CD46 expression (Supplementary Fig. 2b) in ARPE-19 cells caused a significant reduction in TB40/E^FLAG YFP^ and AD169^BADrUL131^ infected cells compared to scrambled siRNA (SCR) (Fig. 4g). Further, AD169^BADrUL131^ and TB40/E wt infected cells knocked-down (KD) for CD46 demonstrated a dramatic decline in IE1 protein levels when compared to controls (Fig. 4h, lanes 4 and 7). To address whether CD46-dependent entry occurs in fibroblasts, CMV infectivity was evaluated with CD46-KD by siRNA in MRC5s and human foreskin fibroblasts (HFF-1) (Supplementary Fig. 2b). No significant inhibition of infection was observed in AD169^BADrUL131^ infected MRC5 and HFF-1 CD46-KD cells (Supplementary Fig. 2c). Importantly, this was confirmed by the observation that anti-CD46 mAbs were unable to limit infection by TB40/E wt and AD169^BADrUL131^ in fibroblasts (Supplementary Fig. 2d). These results implicate the existence of CD46-dependent entry into ARPE-19 cells with no or limited dependency of CD46 for entry in fibroblasts. CMV can enter cells at the cell surface by membrane fusion/macropinocytosis in a pH-independent manner, which occurs mainly in fibroblasts, or within the endosome and/or by macropinocytosis in a pH-dependent manner^14^, which has been demonstrated in epithelial cells^28^. Given our findings, we propose that CD46-dependent virus entry in epithelial cells is through the endocytic pathway, and that CD46 does not play a role in entry of fibroblasts due to the contrasting reliance on fusion or macropiniocytosis at the cell surface.

### CD46 is involved in CMV entry and dissemination

**Fig. 5:**
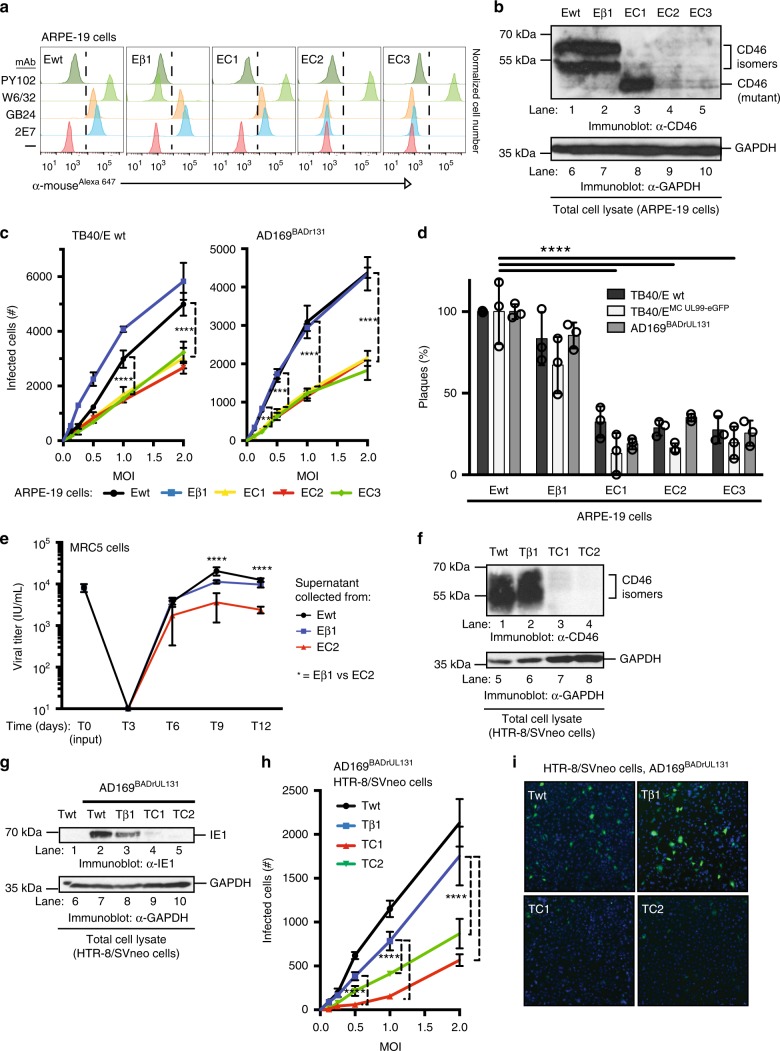
CD46 is an important factor for CMV infection and spread. **a** CD46 cell surface expression of wild-type ARPE-19 cells (Ewt), β_2_m Knock-out (KO) ARPE-19 cells (Eβ1), and three CD46-KO ARPE-19 cell clones (EC1, EC2, and EC3) was assessed by flow cytometry with mAbs against CD46 (2E7 and GB24), MHC class I (W6/32), and PY102 as a non-binding control. Following incubation with anti-mouse IgG^Alexa647^, the normalized cell number was plotted based on Alexa647 fluorescence intensity. **b** The respective KO cells (**a**) were subjected to anti-CD46 and GAPDH immunoblots. **c** ARPE-19 wt and CD46- and β_2_m-KO cells infected with TB40/E wt or AD169^BADrUL131^ were analyzed for infection by an HTI. Total number of infected cells was determined using anti-IE1 antibodies. **d** ARPE-19 wt and CD46- and β_2_m-KO cells infected with TB40/E wt, TB40/E^MC UL99-eGFP^, or AD169^BADrUL131^ were subjected to a plaque assay, counted 14 dpi. **e** Supernatant from Ewt, Eβ1, and EC2 cells infected (E: epithelial cells) with AD169^BADrUL131^ (MOI:0.1) from days 0–12 was added to MRC5 and analyzed for infection at 24 hpi using YFP fluorescent with a cytometer. The virus titre (infectious units (IU)/ml) was plotted for up to 12 dpi. **f** Cell lysates of wt HTR-8/SVneo (Twt) cells, β_2_m-KO HTR-8/SVneo (Tβ1) cells, and CD46-KO HTR-8/SVneo cell clones (TC1 and TC2, T: trophoblasts) were subjected to immunoblot using anti-CD46 and anti-GAPDH antibodies. **g** Cell lysates of AD169^BADrUL131^ (MOI:2) infected Twt, Tβ1, TC1 and TC2 were subjected to anti-IE1 and GAPDH immunoblots. **h** ARPE-19 wt and Tβ1, TC1, and TC2 clones infected with AD169^BADrUL131^ at varying MOIs were analyzed for infection by an HTI assay. The total number of infected cells was determined using a cytometer. **i** Representative cytometer images of (**h**) (overlay: Hoechst stain for nuclear stain (blue) and GFP expression (green) for virally infected cells) (MOI 2). The polypeptides and molecular weight markers are indicated for the immunoblots. Infection experiments (excluding westerns) were performed in triplicate. s.d. is depicted in the experiment. **P < 0.01, ***P < 0.001, ****P < 0.0001 (Student’s two-tailed *t* test)

To further characterize the role of CD46 in CMV infection and dissemination, CD46 was knocked-out (KO) of ARPE-19 cells with CRISRP/Cas9 technology. The expression of CD46 was assessed by flow cytometry (Fig. 5a) and an anti-CD46 immunoblot (Fig. 5b) with confirmation by nucleotide sequencing the targeted cellular genome (Supplementary Fig. 3a). CRISRP/Cas9 was utilized to create two **e**pithelial cell **C**D46-KO clones (EC2 and EC3), one clone with a mutated CD46 (EC1), and a β_2_m-KO control (Eβ1). We initially evaluated TB40/E wt and AD169^BADrUL131^ infection at different MOIs (Fig. 5c). There was a significant reduction in infected cells for both strains in all three CD46-KO populations in comparison to wt, supporting findings with siRNA-treated cells that modulating levels of CD46 negatively impacted virus infection. Importantly, over-expression of CD46 in CD46-KO cells (Supplementary Fig. 3c) was able to restore viral entry (Supplementary Fig. 3d). A predicted alteration of SCR2 splicing due to an edit in exon 3 of clone EC1 (Supplementary Fig. 3a, b) resulted in reduced infectivity equivalent to a complete CD46-KO. MAb GB24 binds to a conformational epitope of CD46 and confirms that this mutant is being expressed at the surface, therefore edits in the SCR2 domain indicate it as the functional region of CD46 in CD46-dependent CMV entry or that partial misfolding of a greater region of this mutant protein has disrupted the function.

Subsequently, virus dissemination was evaluated in the CD46-KO cells by plaque assay with TB40/E wt, the variant TB40/E^MC UL99-eGFP^, and AD169^BADrUL131^ (Fig. 5d). A significant decrease in number of virus plaques was observed in each CD46-KO clone for all virus strains, demonstrating the importance of CD46 in CMV dissemination. In fact, the role of CD46 during viral spread is demonstrated by a 70% decrease in virus plaques (Supplementary Fig. 3e). The multi-cycle viral replication assay further supports the role of CD46 in dissemination (Fig. 5e). A significant decrease in virus titre produced from CD46-KO cells was measured in comparison to wild type (Ewt) and β_2_m-KO control (Eβ1) ARPE-19 cells. These data implicate CD46 as a factor in both CMV infection and dissemination in epithelial cells.

To address the role of CD46 in a cells relevant to congenital CMV^29^, we knocked out CD46 in HTR-8/SVneo cells, trophoblasts immortalized from a 6–12 week gestation placenta, and evaluated CMV infection. CD46 expression in wt HTR-8/SVneo trophoblast cells (Twt), a single clone of β_2_m-KO HTR-8/SVneo cells (Tβ1), and two CD46-KO HTR-8/SVneo clones (TC1 and TC2) was confirmed by immunoblot analysis (Fig. 5f) and nucleotide sequencing of the cellular genome (Supplementary Fig. 3a). A decrease in IE1 protein levels was observed in AD169^BADrUL131^ infected CD46-KO HTR-8/SVneo cells (Fig. 5g, lanes 4–5). Moreover, we evaluated virus infection with different MOIs of AD169^BADrUL131^ and observed a significant inhibition of infected cells in both TC1 and TC2 clones (Fig. 5h). A clear reduction in GFP expression in TC1 and TC2 cells compared to Twt and Tβ1 cells was also apparent in representative images (Fig. 5i). The data support the role of CD46 in CMV infection of trophoblasts. In the context of CD46 as a modulator of compliment activation in the placenta^30^, this presents a complex and critical role for CD46 in both protecting the fetus from immune rejection and facilitating CMV entry.

### CD46 is involved in a viral post-binding entry step

**Fig. 6:**
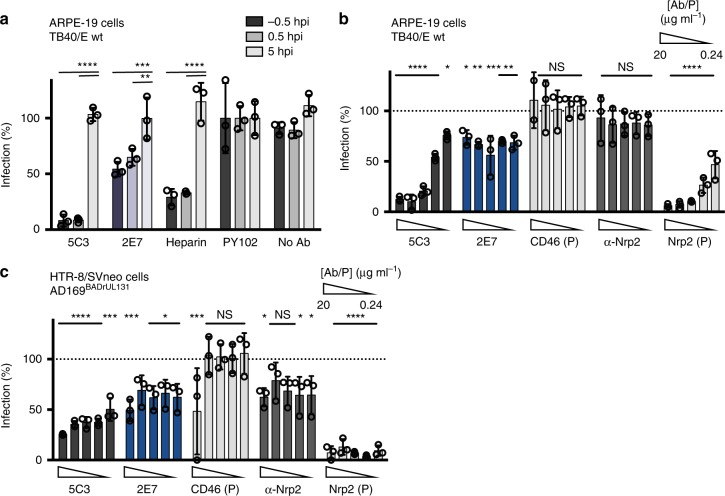
CD46 is involved in a post-binding entry step. **a** TB40/E wt infection of ARPE-19 cells untreated or treated at −0.5, 0.5, and 5hpi with mAbs targeting HCMV gH (5C3) and CD46 (2E7), heparin, a non-binding antibody control (PY102), or a no-antibody control (10 μg ml^−1^) was analyzed with a HTI. PY102-treated cells was used as 100% infection. **b** TB40/E wt infection of ARPE-19 cells and (**c**) AD169^BADrUL131^ infection of HTR-8/SVneo cells incubated with mAbs 5C3, 2E7, and anti-Nrp2 (αNrp2), or soluble CD46 and Nrp2 proteins (P) (20–0.24 μg ml^−1^, in threefold dilutions) were subjected to the HTI assay. The % infection was determined using incubation with PY102 as 100% infection. Experiments were performed in triplicate. s.d. is depicted in the experiment. *P < 0.05, **P < 0.01, ***P < 0.001, ****P < 0.0001 (Student’s two-tailed *t* test)

To determine the step of virus entry impacted by CD46, mAbs 2E7 and 5C3 (anti-gH) and heparin were added to ARPE-19 cells up to 5 h post infection (hpi) with TB40/E wt (Fig. 6a) and AD169^BADrUL131^ (Supplementary Fig. 4a) and evaluated for virus infection. In all cases, 2E7, 5C3 and heparin significantly inhibited virus infection when added prior to infection and 0.5 hpi. The anti-gH mAb 5C3, that blocks entry at a post-binding step,^31^ inhibited virus infection in a similar manner to 2E7. These results suggest that CD46 is involved in a post-viral binding^32^ step of CMV entry and unlikely acts as a viral receptor. Additionally, over-expression of CD46 in ARPE-19 cells has no enhancing effects on CMV infection, which further supports that its involvement is not congruent with acting as a receptor^11^.

To further characterize the role of CD46 compared to the newly discovered Nrp2 receptor during virus infection, soluble CD46 and Nrp2 protein was assessed in epithelial cells and trophoblasts for the ability to neutralize infection in parallel with antibodies targeting the respective proteins (Fig. 6b, c). Soluble Nrp2 protein demonstrated clear reduction of infection for both cell types in comparison to soluble CD46 protein, a result consistent with previous findings^11^. Also as expected, anti-CD46 antibodies limited virus infection in both the epithelial cells and trophoblasts. Yet, anti-Nrp2 mAbs demonstrated a limited effect on virus infection in epithelial cells when compared to trophoblasts. These findings may be explained by potential differences in antibody affinity to Nrp2 expressed on the various cells, a greater affinity of CMV pentamer to Nrp2 than CD46^11^, and/or that CMV infection of trophoblasts is more dependent on Nrp2. These differences highlight the complexity of CMV entry through the contrasting roles for Nrp2 and CD46 (Supplementary Fig. 4b), with Nrp2 playing a receptor-like role and CD46 acting as a factor that participates in an entry step downstream of viral binding to the cell surface.

## Discussion

Our CMV high-throughput inhibition assay in epithelial cells screened a panel of mAbs and identified two clones that limit infection by targeting CD46, but fail to block viral entry of fibroblasts. We propose that CD46-dependent entry is possibly through a post-binding event (Fig. 6, and Supplementary Fig. 4) that involves the interaction of CD46 with the CMV PC. The recently described entry factor Nrp2 acts as a cellular receptor through its engagement with the CMV PC to mediate virus binding to non-fibroblast cell types^11^. In fact, it has been demonstrated that CD46 bound to the PC with low affinity and is consistent with our finding that CD46 may not be a major viral receptor^11^(Fig. 6). Following Nrp2 binding, the virus is endocytosed, where viral envelope then fuses with the cellular membrane in a pH-dependent manner and releases capsid into the cytosol. Given our findings, we propose the model that CD46 likely functions in a post-binding step and that it could impact viral endocytosis and/or virus penetration/fusion during entry. Yet, the results do not completely exclude the role of CD46 as a low affinity modulator to enhance viral binding to Nrp2 or other cellular factors. In addition, the interaction of the PC with CD46 at the cell surface or within the endosome may trigger an activation event causing the phosphorylation of the cytoplasmic tail, a result observed upon binding of *Neisseria to* CD46^33^. The phosphorylation of CD46 and its subsequent activation can induce cytoskeletal rearrangement^34^ and impacting various steps of virus infection from endocytosis to capsid transport. Alternatively, binding of CD46 with the PC may function to induce a conformational change of the viral/cellular protein complex that promotes endocytosis or possibly activate the fusogenic viral complex to induce viral penetration/fusion. Furthermore, the reduced CMV dissemination in CD46-KO epithelial cells suggests that viral spread and egress in epithelial cells may occur in a CD46 dependent process. CD46 may direct the trafficking of viral envelope complexes to sites of assembly or regulate the exocytosis process of mature virions. Collectively, these findings demonstrate that CD46 is a cellular factor that participates in both CMV entry and dissemination in epithelial cells.

The function of CD46 during infection extends beyond its expression on the cell surface and importantly, CD46 expression alone does not confer dependency of virus entry. Fibroblast cell lines (MRC5 and HFF-1) express CD46 but do not show inhibition of infection when CD46 is targeted with mAbs or knocked down with siRNA (Supplementary Fig. 2). CD46 likely engages virus through an interaction with the viral pentamer and may play a role during viral endocytosis and the occurrence of this pathway is the possible determinant of CD46 importance. The interaction of CD46 with one or several known entry factors (e.g., PDFGRα, Nrp2, CD147, and/or OR14I1) or yet to be identified factors may be required for virus infection by mediating viral endocytosis or viral fusion with the endosomal membrane. Also, the role of CD46 in viral entry of epithelial cells and trophoblasts may be dependent on intracellular signaling molecules following virus interaction with cell-type specific CD46 protein-containing complexes that mediate downstream infection steps. Only upon identifying and exploring CD46-interacting factors in epithelial cells and trophoblasts that participate in virus entry can the molecular function of CD46 be fully elucidated. Interestingly, CMV also enters endothelial cells by the endocytic pathway and thus CD46 probably plays a role in infection of endothelial cells. Collectively, cell-type specific CD46-protein containing complexes would likely be important during unique steps of virus entry in cells that require endocytosis for infection.

Placental defects observed in congenital CMV can be attributed to CMV infection of placental cytotrophoblasts during early gestation that deregulates multiple signaling pathways vital for placental development^35^. CD46 expression is reported within placental tissues in both differentiated syncytiotrophoblast epithelium and villous cytotrophoblasts, with CD46 expression decreasing amid the maturation of villous cytotrophoblasts into extra-villous cytotrophoblast cell columns^30,36^. Interestingly, high levels of CD46 expression can be observed in HTR-8/SVneo trophoblast cells in comparison to HEK293T, MRC5 and ARPE-19 cells (Supplementary Fig. 2e). Our findings that CMV infection is limited in CD46-KO trophoblasts indicate that CD46-dependent entry may occur in congenital CMV. Notably, it has been demonstrated that anti-CD46 mAbs targeting CD46 domain SCR1 do not block complement binding to CD46^37^, and are therefore potential congenital CMV therapeutics that can inhibit CMV infection while not inhibiting the CD46 complement inhibitory function. In summary, high levels of CD46 may be responsible for both protecting the fetus from complement activation and facilitating congenital CMV infection.

Screening for novel cellular factors necessary for virus entry by utilizing a library of mAbs offers many advantages over traditional genetic screening approaches (e.g., siRNA, CRISPR/Cas9) because it does not manipulate transcriptional profiles and eliminates the toxic effects of transfection and transduction. The flexibility to customize this screen for different viruses and diverse cell types adds to its relevance. Additionally, the identified mAbs can be utilized as tools for further study of entry and a platform for the development of potential therapeutics. The ability to tailor this screen to different cell types has the added potential of discovering biomarkers consistent with CMV infection, and could be applied to cancer cells for initiating cancer specific response. Ultimately, our results present an immunization and screening strategy that has identified CD46 as a CMV entry factor and expands the molecular requirement of CMV entry.

## Methods

### Cells, antibodies, and viruses

Human retinal epithelial ARPE-19 cells (ATCC #CRL-2302) were cultured in a 1:1 ratio of Dulbecco’s modified Eagle’s medium (DMEM) to Kaighn’s modification of Ham’s F-12 medium (F-12k). A549 human lung epithelial cells (ATCC #CCL-185) were cultured in F-12k. Jurkat, Clone E6-1 (ATCC #TIB-152) peripheral blood T lymphocytes and HTR-8/SVneo (ATCC #CRL-3271) were cultured in RPMI-1640 medium. MRC5 lung fibroblasts (ATCC #CCL-171), HFF-1 (ATCC SCRC-1041), and HEK293T (ATCC #CRL-3216) were cultured in DMEM. All cell lines were supplemented with 10% fetal bovine serum (FBS), 1 mM HEPES, 100 U ml^−1^ penicillin, and 100 g ml^−1^ streptomycin at 37 °C in a humidified atmosphere (5% CO_2_). Anti-CD46 immunoglobulins were raised in rabbits by inoculation with aa36–140 of CD46 (GenScript, Piscataway, NJ). The CMV anti-immediate early-1 (IE1) immunoglobulins were raised in rabbit by inoculation with a peptide derived from the CMV TB40/E sequence shared by the IE1 and IE2 protein^38^ (GenScript). The CMV neutralizing gH mAb 5C3 was purified from hybridoma culture supernatant^31^. The mAb PY102, utilized as a non-binding control, recognizes the globular head of the HA of PR/8 (H1N1) influenza A virus.^39^ The mAb W6/32, which recognizes properly folded MHC class I molecules, was purified from hybridoma culture supernatant. Anti-CD46 mAbs TRA-2–10^26^ and GB24^27^ were gifts from Dr. J P Atkinson (Washington University, St. Louis, MO). Anti-CD90 mAb 5E10 (Thermo Fisher), anti-glyceraldehyde-3-phosphate dehydrogenase (GAPDH) (Chemicon) and anti-Nrp2 (Thermo Fisher-257103) were purchased. The production of mAbs 2E7, 12H8, and anti-gB ITC88 is described below.

The clinical CMV isolate TB40/E (wt) and the bacterial artificial chromosome (BAC) clone TB40-BAC4 were kind gifts of Dr. Christian Sinzger (University of Medical Center Ulm) and Dr. Felicia Goodrum (University of Arizona). TB40/E wt, the recombinant virus TB40/E^FLAG YFP^ ^23^ (constructed from TB40-BAC4), the AD169 strain BADrUL131 C4^15^ with repaired UL131 ORF and expresses GFP (Dr. Thomas Shenk, Princeton University), TB40/E-*mCherry*-UL99*eGFP* strain^40^ (Dr. Eain A. Murphy, Cleveland Clinic) and the UL32-EGFP-HCMV^41^ (Dr. Christian Sinzger) were propagated in MRC5 cells. Virus from infected-cell supernatant and cell lysates were pooled, sonicated, and purified by density gradient centrifugation at 21 k rpm (10°, 90 min, 20% sorbitol cushion)^42^. Infectious virus yield was assayed on MRC5 cells by medium tissue culture infective does (TCID_50_).

### Generation of mAb library against ARPE-19-derived vesicles

ARPE-19 cells (120 million) were subjected to two rounds of freeze-thaw and sonicated in bath sonicator (15 min). Then debris was pelleted (5000 × *g*) and discarded. The supernatant was ultracentrifugated (20,000 r.p.m, SW28 (~50,000 × *g*)) with a sorbitol cushion to isolate membrane fraction followed by sonication to clarify. Mice were immunized with the ARPE-19-derived membrane fraction (50 μg), followed by two boosts every 3 weeks. ARPE-19 cells were incubated with sera from immunized mice at 1:100, 1:1000, and 1:10,000, washed 1×, incubated with goat anti-mouse conjugated with Alexa-647 (4 μg ml^−^^1^) and analyzed for antibodies against the cell surface proteins of ARPE-19 cells with an Intellicyt HTFC flow cytometer (Intellicyt Inc.). The data was analyzed using Flow Jo software (Tree Star, Inc). The ability of the serum from immunized mice to inhibit a CMV infection was performed at two dilutions (1:100, 1:1000) with TB40/E^FLAG YFP^ (MOI:5) infected ARPE-19 cells (1 h 4 °C). Virus-infected cells were determined using an Acumen ^*e*^X3 laser-scanning fluorescence microplate cytometer that measured YFP fluorescence at 16 h post infection (hpi). B-cell hybridomas were generated from the spleen of the selected by the Center for Therapeutic Antibody Development (CTAD). The individual B-cell clones were selected from soft agar by a robotic Hamilton/Stem Cell Technology ClonaCell Easy Pick instrument in order to generate single-cell hybridomas.^26^ All animal experiments were performed in accordance with the Icahn School of Medicine at Mount Sinai Institutional Animal Care and Use Committees (IACUC).

### Transmission electron microscopy imaging/vesicle analysis

Vesicle preparations (2 mL) were applied onto glow discharged carbon coated copper grids and negatively stained using a previously procedure^43^. Specimens were imaged with a JEM-1400 (JEOL, USA) TEM equipped with an ultrascan 1000XP 2Kx2K PIXEL CCD CAMERA (Gatan, UK). Particle selection and measurement were performed with the program ImageJ. The distribution of vesicle diameters was determined by averaging two measurements of each vesicle (*n* = 29) with ImageJ and the calculated conversion of 2.6 A per pixel.

### High-throughput infectivity assay (HTI)

Monoclonal antibody library screen: Hybridoma supernatant from clones was screened for their ability to inhibit TB40/E^FLAG YFP^ (MOI:3) infection of ARPE-19 cells^31^. Virus was pre-incubated with hybridoma supernatant (1 h 4 °C), added to ARPE-19 cells (1 × 10^4^ per well in a 96-well) for 2 h @ 37 °C, and then replaced with new media. Virus-infected cells were analyzed for YFP fluorescence intensity at 16hpi with an Acumen ^*e*^X3 laser-scanning fluorescence microplate cytometer. The number of YFP positive cells was used to calculate % infection using media alone as 100%.

### Monoclonal antibody inhibition assay

Virus (MOI:0.5) incubated with respective mAbs (2 μg ml^−1^, or otherwise noted) or soluble proteins Nrp2 (P) and CD46 (P) (Sino Biological (cat#: 10695-H08H and 12239-H08H) for 1 h 4 °C was used to infect ARPE-19, HTR-8/SVneo, or MRC5 cells (1 × 10^4^ cell/well in a 96-well plate) for 2 h (37 °C) and then replaced with new media. At 24 or 48 hpi, cells fixed with 4% paraformaldehyde were either directly analyzed for YFP or GFP if a reporter virus was used or probed for CMV IE1 protein (see below: Immunostain assay). Cells were also stained with Hoechst 33342 (1 μg ml^−1^, Molecular Probes) in PBS (15 min room temperature). The Celigo Image Cytometer (Nexcellom Bioscience) was utilized to analyze fluorescence intensity from cells to determine virus infection. Virus infection (%) was determined relative to non-binding mAb control PY102. Note, similar dose dependent antibody concentration was observed with both TB40/E wt (Fig. 4a) and AD169^BADrUL131^ (unpublished data).

### Sequencing of immunoglobulin in B cell hybridoma clones

RNA was extracted from hybridoma cell pellets (<5 × 10^6^ cells) using an RNeasy RNA extraction kit as per manufacturer’s instruction (Qiagen). Approximately, 500 ng of hybridoma RNA was then used as a template for first strand cDNA conversion and 5′ RACE PCR with the 5′ SMARTer RACE kit (TaKaRa/Clontech). To facilitate the cDNA conversion and 5′ RACE, an external 3′ gene specific primer (GSP1) and internal 3’ primer (GSP2) were designed, based on conserved regions within the mouse IgG1 CH1 constant domain: mG1_GSP1 (5′-AGAGGTCAGACTGCAGGACA-3′) and G1_GSP2 (5′-CCCAGGGTCACCATGGAGTT-3′). PCR product was submitted for Sanger sequencing utilizing the GSP2 primer (GeneWiz) and sequences were blasted against the IMGT database for antibody characteristics and junction analysis (www.imgt.org). Variable sequence was then submitted for gene block synthesis (Integrated DNA Technologies, Skokie, IL) with added 5′ and 3′ restriction sites corresponding to in-frame restriction sites in antibody generating cloning vectors, pFUSE_CHIg and pFUSE_CLIg, (InVivoGen, San Diego, CA), each of which contain mouse heavy and light chain constant genes for mouse IgG1 and mouse kappa, respectively, as well as a signal sequence in-frame with the multi-cloning site.

### Cloning and production of anti-gB mAb

For the anti-gB mAb ITC88, human mRNA/cDNA sequence representing the heavy and light chain variable segments of the heterohybridoma ITC88 was obtained from Genbank (https://www.ncbi.nlm.nih.gov), accession numbers, L37310 and L37311, respectively. Coding sequence for the heavy and light variable segments (V-D-J and V-J segments respectively) were then synthesized into gene blocks as above (Integrated DNA Technologies). Heavy and light variable gene block segments were then cloned under standard molecular technique into appropriate heavy and light pFuse vectors, and transformed into DH5α E.coli. Colonies were picked and screened by PCR for variable inserts. Ten positive clones were grown and the isolated DNA (Qiagen) was sequencing using vector specific primers. Plasmids encoding for confirmed heavy and corresponding light chain were transfected in a 1:1 ratio into Expi293F cells (ThermoFisher) using Lipofectamine 2000 and supernatants were collected after 5 days for secreted mAb.

### Metabolic labeling of cells

ARPE-19 cells (5 million) were metabolically labeled with ^35^S-methionine for 6 h at 37 °C. Cells were lysed in 0.5% NP40 lysis mix^26^ and immunoprecipitated using mAbs 2E7 and 12H8. Following incubation of immune complexes with protein G-agarose beads (RepliGen), the recovered polypeptides were resolved on a SDS-polyacrylamide gel (12.5%) and visualized using fluorography^26^.

### Mass spectroscopy proteomic analysis

For identifying the target protein of mAb 2E7, ARPE-19 cells (10 million) lysed with 0.5% NP40 lysis mix^26^ were incubated with 2E7 mAb pre-bound to protein G-agarose beads (RepliGen) for 6 h at 4 °C. The recovered proteins were resolved on a SDS-polyacrylamide gel (12.5%) and visualized using Gel Code Blue Reagent (Thermo Fisher). Polypeptides migrating at 60 kDa and 50 kDa were excised and subjected to mass spectrometry proteomic analysis at Bioproximity, Inc (Chantilly, VA)^31^.

### Immunoprecipitation and western blot

Immunoprecipitation (IP) experiments were performed by incubating cells lysates (0.5% NP40 lysis mix) with the respective antibody followed by protein A or G-agarose beads. The recovered proteins were resolved on an SDS-polyacrylamide gel (12.5%). For analysis of total cell lysates, cells were lysed in 1%SDS (in PBS) with three rounds of heating (95 °C)/scratching tube against a metal test tube rack and quantified using a Pierce BCA protein assay kit (Thermo Scientific). Equal amount of proteins (20–30 μg) were separated on a SDS-polyacrylamide gel (12.5%). The analysis of IE1 proteins required additional (3×) protein lysates. The proteins from the gels were then transferred to a PVDF membrane (0.45μm, Thermo Scientific) and the membrane was incubated in 5% non-fat drymilk/0.01% Tween-20/0.02% sodium azide in PBS followed by a primary antibody (anti-CD46 (2 μg ml^−1^)), anti-IE1 (2 μg ml^−1^) or anti-GAPDH (0.1 μg ml^−1^) Millipore) and anti-rabbit or anti-mouse conjugated to HRP (1:20,000 or 1:10,000, respectively). Uncropped and unprocessed scans are included in the Source Data file.

### Immunostain assay for CMV IE1 protein

Virus-infected cells at 24 or 48 hpi were fixed with 4% paraformaldehyde (30 min RT), permeabilized with 0.3% Triton X-100 in PBS and blocked with 4% normal goat serum (NGS). The cells were probed with rabbit anti-IE1 antibody (2 μg ml^−1^) followed by chicken anti-rabbit Ig conjugated with Alexa647 (1:1000) (Thermo Fisher) and analyzed by a Celigo Image Cytometer for fluorescence intensity.

### Pre/post binding inhibition assay

ARPE-19 cells (1 × 10^5^/well in a 96-well plate) infected with TB40/E wt or AD169^BADrUL131^ (MOI:0.5) were incubated anti-gH mAb 5C3, anti-CD46 mAb 2E7, heparin (used as a classical method for blocking binding) and a non-binding control PY102 (10 μg ml^−1^) for 30 min at −0.5 hpi and at 0.5 hpi with cells always on ice. The cells were then transferred to 37 °C to allow virus infection. As a control, mAbs/heparin were added 5 hpi. The infected cells were fixed with 4% paraformaldehyde at 24 hpi and analyzed for virus infection by the Celigo Image Cytometer for GFP infection (AD169^BADrUL131^) or immunostain for IE1 expression (TB40/E wt). The % infection was determined relative to the non-binding mAb control PY102.

### Flow cytometry analysis

The respective cells were blocked with 1%BSA/PBS (4 °C), incubated with primary antibody (2 μg ml^−1^) or hybridoma supernatant at specified dilutions followed by goat anti-mouse conjugated with Alexa647 (4 μg ml^−1^)(ThermoFisher Scientific) and analyzed using an Intellicyt HTFC flow cytometer (Intellicyt). For analysis of permeabilized cells, cells were fixed in BD Biosciences Cytofix/Cytoperm solution (20 min 4 °C) and maintained in 0.1% saponin.^44^ The data was analyzed using Flow Jo software (Tree Star, Inc) and the mean fluorescence intensity (MFI) was determined for each sample. For Figs 1d, 4d, 5a and Supplementary Figs 2b and 3f, the normalized cell number was plotted based on Alexa647 fluorescence intensity. For analysis of GFP or YFP fluorescent intensity from a respective reporter virus, cells could be directly analyzed with the HTFC flow cytometer. For the analysis of the mAb library (Fig. 2), fold change was determined relative to MFI of the non-binding anti-CMV gH control (5C3). The heat-map was generated using Morpheus (https://software.broadinstitute.org/morpheus/).

### mAb binding competition experiment

ARPE-19 cells blocked with 1%BSA/PBS were incubated with mAb 2E7 conjugated with Alexa647 (2 μg ml^−1^) with increasing amount of non-labeled mAb 2E7, TRA-2-10, GB24, and PY102 and analyzed with the HTFC flow cytometer. The data was analyzed using Flow Jo software (Tree Star, Inc).

### siRNA infection assay

ARPE-19, MRC5, and HFF-1 cells (2 × 10^5^/well in a 6-well plate) were transfected with SMARTpool: siGENOME CD46 siRNA or siGENOME Non-Targeting siRNA pool #1 (20 nM) (Dharmacon) by RNAiMAX (ThermoFIsher) according to manufacturer’s instructions. 48 h post transfection, cells were infected with TB40/E^FLAG YFP^ or AD169^BADrUL131^ (MOI:0.5) and subjected to flow cytometry or western blotting analysis at 48 hpi.

### CRISPR/Cas9 knock out of cells

Generation of CD46 and β2M knock-out cells: The Target Guide Sequence Cloning Protocol provided by Genome-scale CRISPR Knock-out (GeCKO) and ZhangLab^45^ was utilized to construct pLentiCRISPRv2-CD46 and pLentiCRISPRv2-β2M. Oligos were designed based on a CD46 sgRNA sequence from the GeCKOv2 Human Library B (AACTCGTAAGTCCCATTTGC)^45^ and the crB2M_6 sequence (GGCCGAGATGTCTCGCTCCG).^46^ HEK293T cells were seeded at 5 × 10^5^ per well in a 6-well plate and transfected with 1.8 μg pLentiCRISPRv2-CD46 or pLentiCRISPRv2-β2M, 1.4 μg packaging vector psPAX2 and 0.4 μg envelope vector pCMV-VSV-G (gift from Dr. Benhur Lee, Icahn School of Medicine at Mount Sinai) by Lipofectamine 2000 (Thermo Fisher Scientific). After incubation for 72 h, ARPE-19 or HTR-8/SVneo cells (2 × 10^5^ per well in a 6-well plate) were transduced with filtered (0.45 μm) supernatant diluted (1:1) in DMEM/F-12 k (1:1) media and polybrene (8 μg ml^−1^). Media containing puromycin (2 μg ml^−1^) was added 24 h post transduction to eliminate non-transduced cells and passaged 1:3 for 2 weeks. Surface expression of CD46 and MHC class I molecules were analyzed using anti-CD46 mAb 2E7 and anti-MHC1 mAb W6/32 by flow cytometry. A β_2_m knock out (KO) inhibits correct MHC class I folding, thus limiting the surface expression of a type I membrane protein as a control and does not have an effect on CMV entry. Clones were isolated by serial dilution. Nucleotide sequencing of the respective genome within the targeted genome was facilitated by the InFusion HD Cloning Plus (Takara) and primers (HindIII-CD46-F AGGGAGACCCAAGCTTTGCCTGGGTGAATATGAATC, XhoI-CD46-R TAGATGCATGCTCGAGACTATATATCTTGCCTATCTC, HindIII-B2M-F AGGGAGACCCAAGCTTCAAACTCACCCAGTCTAGT, and Xho-1B2M-R TAGATGCATGCTCGAGTTAAGTAGTCGCGCGTCC). EC2, EC3, TC1, TC2, Eβ1 and Tβ1 clones had insertions and deletions that caused premature stop codons within the cellular genome, while a deletion in the EC1 clone affected a putative splicing site that yielded a truncated CD46 species.

Rescue assay: Eβ1 and EC2 cells were electroporated using the Amaxa SF cell line 4D-Nucleofecter X Kit L system (Lonza) with setting DN-100 Vero cells with CD46 variant c (BC1) cDNA in pCDNA3.1+ (GenScript) and empty vector control pCDNA3.1+. CD46 expression in the cells was evaluated by immunoblot analysis. For evaluating infectivity, electroporated Eβ1 and EC2 cells were infected (in triplicate) with TB40/E^FLAG YFP^ and AD169^BAD*r*UL131^ (MOI:0.5) 24 h post electroporation. Following infection (1 h 37 °C), the % of YFP/GFP positive cells was determined by flow cytometry at 24 hpi and plotted as total.

### Plaque reduction assay

ARPE-19 wt, Eβ1, EC1, EC2, and EC3 cells (5 × 10^4^ cells per well in a 24-well plate) in DMEM/F12k (1:1) were infected in triplicate with TB40/E wt, TB40/E^MC UL99*eGFP*^ and AD169^BAD*r*UL131^ at varying MOIs (MOI: 0.01, 0.005, 0.001). Following infection (1 h 37 °C), inoculum was removed and replaced with an agarose overlay of 2.5% SeaPlaque Agarose (Lonza) and DMEM/F12k (1:1) media at a 2:3 ratio. Once solidified, additional media was added to each well. At 7–10 dpi, cells were fixed with 4% paraformaldehyde (30 min room temperature). The plates were analyzed for a viral plaques using the Celigo Image Cytometer (Nexcellom Biosciences) for aggregation of YFP or RFP fluorescence and images captures were utilized to blind count number of plaques. For TB40/E wt, cells were stained with crystal violet (0.1% crystal violet/10%ETOH) stain and plaques were counted using a wide-field light microscope.

### Multi-cycle replication assay

ARPE-19 wt, Eβ1, and EC2 cells were seeded with DMEM/F12k (1:1) media at 5 × 10^4^ cells per well in a 24-well plate (BD-Falcon). 24 h later, the cells were infected in duplicate per time-point with virus strain AD169^BAD*r*UL131^ at MOI: 0.01. Supernatant collected immediately (Day 0), Day 3 (T3), Day 6 (T6), Day 9 (T9) and Day 12 (T12) was seeded on to MRC5 cells (1 × 10^4^ cell/well) in a 96-well plate. After incubation for 2 h (37 °C), inoculum was then replaced with new media. At 24hpi, cells were fixed in 4% paraformaldehyde, stained with Hoechst reagent, and number of virus-infected cells was determined using fluorescence intensity with a Celigo Image Cytometer (Nexcellom Bioscience). The number of GFP positive cells/ml of cell supernantant was used to determine the virus titre as infectious units per mililiter of supernatant (IU/ml).

### Statistical analysis

All data points were derived from triplicate samples. The Student’s unpaired, two-tailed *t* tests were calculated in GraphPad Prism (La Jolla, CA), with one asterisk (*) representing statistical significance of *P* < 0.05 (**P < 0.01, ***P < 0.001, ****P < 0.0001). In all relevant figures, standard deviation is depicted.

### Reporting summary

Further information on research design is available in the Nature Research Reporting Summary linked to this article.

## Supplementary information

Supplementary Information

Reporting Summary

Source Data

## Data Availability

The source data underlying all figures are provided as a Source Data file.

## References

[1] Herndler-Brandstetter D, Almanzar G, Grubeck-Loebenstein B (2006). Cytomegalovirus and the immune system in old age. Clin. Appl. Immunol. Rev..

[2] Boeckh, M. & Geballe, A. P. Science in medicine Cytomegalovirus: pathogen, paradigm, and puzzle. *Am. Soc. Clin. Investig*.**121**, 1673–1680 (2011). 10.1172/JCI45449 PMC3083799 21659716

[3] Syggelou A, Iacovidou N, Kloudas S, Christoni Z, Papaevangelou V (2010). Congenital cytomegalovirus infection. Ann. N. Y. Acad. Sci..

[4] Söderberg-Nauclér C (2006). Does cytomegalovirus play a causative role in the development of various inflammatory diseases and cancer?. J. Intern. Med..

[5] Compton T, Nowlin DM, Cooper NR (1993). Initiation of Human Cytomegalovirus Infection Requires Initial Interaction with Cell Surface Heparan Sulfate. Virology.

[6] Feire AL, Koss H, Compton T (2004). Cellular integrins function as entry receptors for human cytomegalovirus via a highly conserved disintegrin-like domain. Proc. Natl Acad. Sci. USA.

[7] Wang X, Huong SM, Chiu ML, Raab-Traub N, Huang ES (2003). Epidermal growth factor receptor is a cellular receptor for human cytomegalovirus. Nature.

[8] Chan G, Nogalski MT, Yurochko AD (2009). Activation of EGFR on monocytes is required for human cytomegalovirus entry and mediates cellular motility. Proc. Natl Acad. Sci. USA.

[9] Soroceanu L, Akhavan A, Cobbs CS (2008). Platelet-derived growth factor-α receptor activation is required for human cytomegalovirus infection. Nature.

[10] Li Q, Wilkie AR, Weller M, Liu X, Cohen JI (2015). THY-1 cell surface antigen (CD90) has an important role in the initial stage of human Cytomegalovirus infection. PLoS Pathog..

[11] Martinez-Martin N (2018). An unbiased screen for human Cytomegalovirus identifies neuropilin-2 as a central viral receptor. Cell.

[12] Vanarsdall AL (2018). CD147 promotes entry of pentamer-expressing human cytomegalovirus into epithelial and endothelial cells. MBio.

[13] E X (2019). OR14I1 is a receptor for the human cytomegalovirus pentameric complex and defines viral epithelial cell tropism. Proc. Natl Acad. Sci. USA.

[14] Gardner TJ, Tortorella D (2016). Virion glycoprotein-mediated immune evasion by human Cytomegalovirus: a sticky virus makes a slick getaway. Microbiol. Mol. Biol. Rev..

[15] Wang D, Shenk T (2005). Human cytomegalovirus virion protein complex required for epithelial and endothelial cell tropism. Proc. Natl Acad. Sci. USA.

[16] Wussow F, Chiuppesi F, Contreras H, Diamond D (2017). Neutralization of human cytomegalovirus entry into fibroblasts and epithelial cells. Vaccines.

[17] Kabanova A (2016). Platelet-derived growth factor-α receptor is the cellular receptor for human cytomegalovirus gHgLgO trimer. Nat. Microbiol..

[18] Ryckman BJ (2008). Characterization of the human cytomegalovirus gH/gL/UL128-131 complex that mediates entry into epithelial and endothelial cells. J. Virol..

[19] Vanarsdall AL, Wisner TW, Lei H, Kazlauskas A, Johnson DC (2012). PDGF receptor-α does not promote HCMV entry into epithelial and endothelial cells but increased quantities stimulate entry by an abnormal pathway. PLoS Pathog..

[20] Liszewski MK, Kemper C, Price JD, Atkinson JP (2005). Emerging roles and new functions of CD46. Springe. Semin. Immunopathol..

[21] Wang D, Yu Q-C, Schröer J, Murphy E, Shenk T (2007). Human cytomegalovirus uses two distinct pathways to enter retinal pigmented epithelial cells. Proc. Natl Acad. Sci. USA.

[22] Beletskii A, Galloway A, Rele S, Stone M, Malinoski F (2014). Engineered PRINT nanoparticles for controlled delivery of antigens and immunostimulants. Hum. Vaccin. Immunother..

[23] Noriega VM (2014). Human cytomegalovirus US28 facilitates cell-to-cell viral dissemination. Viruses.

[24] Post BTW (1991). Membrane cofactor protein of the complement system: alternative splicing of serine/threonine/prohne-rich exons and cytoplasmic tails produces multiple isoforms that correlate with protein phenotype. J. Exp. Med.

[25] Wang D, Shenk T (2005). Human cytomegalovirus UL131 open reading frame is required for epithelial cell tropism. J. Virol..

[26] Andrews PW (1985). A human cell‐surface antigen defined by a monoclonal antibody and controlled by a gene on human chromosome 1. Ann. Hum. Genet..

[27] Hsi, Bae-li, Yeh, Chang-jing, G., Patrick, F., Samson, M. & Grivaux, C. Monoclonal antibody GB24 recognizes a Trophoblast-Lymphocyte Cross-Reactive Antigen. *Am. J. Reprod. Immunol. Microbiol*. **18**, 21–27 (1988). 10.1111/j.1600-0897.1988.tb00228.x 2462369

[28] Bodaghi B (1999). Entry of human cytomegalovirus into retinal pigment epithelial and endothelial cells by endocytosis. Invest. Ophthalmol. Vis. Sci..

[29] Chou D, Ma Y, Zhang J, McGrath C, Parry S (2006). Cytomegalovirus infection of trophoblast cells elicits an inflammatory response: A possible mechanism of placental dysfunction. Am. J. Obstet. Gynecol..

[30] Holmes CH (1992). Complement regulatory proteins at the feto-maternal interface during human placental development: distribution of CD59 by comparison with membrane cofactor protein (CD46) and decay accelerating factor (CD55)*. Eur. J. Immunol..

[31] Gardner TJ (2016). Functional screening for anti-CMV biologics identifies a broadly neutralizing epitope of an essential envelope protein. Nat. Commun..

[32] Kim JH, Collins-McMillen D, Caposio P, Yurochko AD (2016). Viral binding-induced signaling drives a unique and extended intracellular trafficking pattern during infection of primary monocytes. Proc. Natl Acad. Sci. USA.

[33] Lee SW (2002). CD46 is phosphorylated at tyrosine 354 upon infection of epithelial cells by Neisseria gonorrhoeae. J. Cell Biol..

[34] Yamamoto H, Fara AF, Dasgupta P, Kemper C (2013). CD46: The ‘multitasker’ of complement proteins. Int. J. Biochem. Cell Biol..

[35] Warner, J. A. et al. Human cytomegalovirus infection inhibits CXCL12-mediated migration and invasion of human extravillous cytotrophoblasts. (2012). 10.1186/1743-422X-9-255 10.1186/1743-422X-9-255 PMC3545970 23116176

[36] Hsi BL, Hunt JS, Atkinson JP (1991). Differential expression of complement regulatory proteins on subpopulations of human trophoblast cells. J. Reprod. Immunol..

[37] Cho S‐W, Oglesbyo TJ, Hsi B‐L, Adams EM, Atkinson JP (1991). Characterization of three monoclonal antibodies to membrane co‐factor protein (MCP) of the complement system and quantification of MCP by radioassay. Clin. Exp. Immunol..

[38] Møller, R. et al. miRNA-mediated targeting of human cytomegalovirus reveals biological host and viral targets of IE2. *Proc. Natl. Acad. Sci. USA* 201719036 (2018). 10.1073/pnas.1719036115 10.1073/pnas.1719036115 PMC5798380 29339472

[39] Reale MA (1986). Characterization of monoclonal antibodies specific for sequential influenza A / PR / 8 / 34 virus variants. J. Immunol..

[40] O’Connor CM, Nukui M, Gurova KV, Murphy EA (2016). Inhibition of the facilitates chromatin transcription (FACT) complex reduces transcription from the HCMV MIEP in models of lytic and latent replication. J. Virol..

[41] Sampaio KL, Cavignac Y, Stierhof Y-D, Sinzger C (2005). Human cytomegalovirus labeled with green fluorescent protein for live analysis of intracellular particle movements. J. Virol..

[42] Gardner TJ (2013). Development of a high-throughput assay to measure the neutralization capability of anti-cytomegalovirus antibodies. Clin. Vaccin. Immunol..

[43] Vink Martin, Derr KD, Love James, Stokes DavidL, I. U.-B. (2005). A high-throughput strategy to screen 2D crystallization trials of membrane proteins. J. Struct. Biol..

[44] Noriega VM (2012). Human cytomegalovirus US3 modulates destruction of MHC class I molecules. Mol. Immunol..

[45] Shalem O (2014). Genome-scale CRISPR-Cas9 knockout screening in human cells. Science.

[46] Mandal, P. et al. Efficient ablation of genes in human hematopoietic stem and effector cells using CRISPR/Cas9. **80**, 631–637 (2013). 10.1016/j.stem.2014.10.004 PMC4269831 25517468

[47] Reynaud JM, Horvat B (2013). Animal models for human herpesvirus 6 infection. Front. Microbiol..

