# Supplementary material for: CD46 facilitates entry and dissemination of human cytomegalovirus
Source: Nat Commun. 2019 Jun 20;10:2699. doi: 10.1038/s41467-019-10587-1 (PMC6586906; doi:10.1038/s41467-019-10587-1)
Supplement: Supplementary file 1 — Supplementary Information [file 41467_2019_10587_MOESM1_ESM.pdf]

a

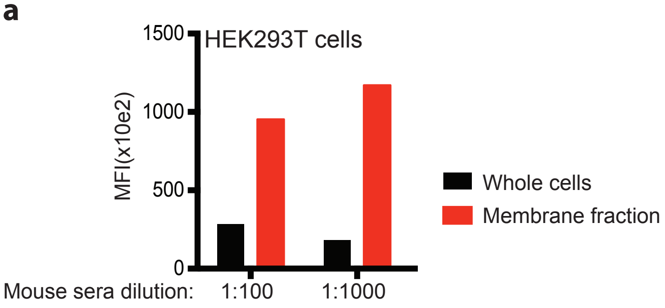

b

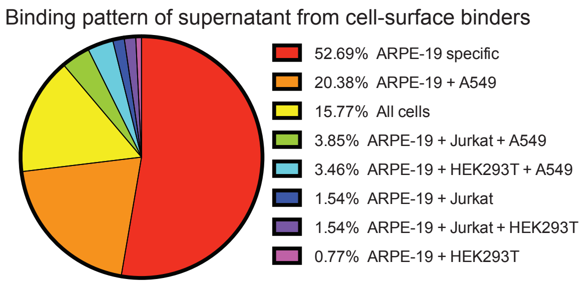

c

mAb 2E7/12H8 sequence

Chain	VH	DH	JH	Junction (CDR3)
Heavy	Musmus IGHV5-17*02 F	Musmus IGHD2-10*02 F	Musmus IGHJ4*01 F	CVRKRYENYGYYGLDYW
Kappa	Musmus IGKV8-19*01 F		Musmus IGKJ5*01 F	CQNDYSYPLTF

d

Mass Spec from ~60kDa polypeptide

Protein Name	Gene name	Description	E-value	Intensity	Peptides	Identifications	Length	Coverage (%)
V9HWE1	HEL113	Epididymis luminal protein 113	-183.73	9.50546	18	47	466	44.2
Q9NNW2	CD46	Membrane cofactor protein	-13.523	8.07879	2	2	392	5.9
B4DNK4	PKM	Pyruvate kinase	-11.7788	7.63009	2	2	457	7.2
Q15233	NONO	Non-POU domain-containing octamer-binding protein	-7.53521	8.04246	2	2	471	5.5

Mass Spec from ~50kDa polypeptide

Protein Name	Gene name	Description	E-value	Intensity	Peptides	Identifications	Length	Coverage (%)
Q8N850	VIM	Vimentin	-532.851	9.65591	33	123	466	69
P13645	KRT10	Keratin, type I cytoskeletal 10	-297.469	9.45608	23	82	584	36.6
P08729	KRT7	Keratin, type II cytoskeletal 7	-86.4138	8.45565	7	16	469	13.43283653
D6RHD5	ALB	Serum albumin	-74.6357	9.52318	6	17	459	13.72549087
P07437	TUBB	Tubulin beta chain	-39.0045	8.30972	6	12	444	18.46846789
K7EQQ3	KRT9	Keratin, type I cytoskeletal 9	-62.9826	8.76826	5	15	390	22.82051295
B4DQV1		cDNA FLJ58994, highly similar to Collagen alpha-1(VI) chain	-57.0192	8.34798	5	15	404	19.3069309
P02533	KRT14	Keratin, type I cytoskeletal 14	-52.3588	8.60361	5	13	472	12.92372942
Q15233	NONO	Non-POU domain-containing octamer-binding protein	-43.0532	8.47997	5	11	471	13.80042434
Q96E76	PKM	Pyruvate kinase PKM	-255.177	9.22132	4	49	531	9.227871895
Q7Z3Y8	KRT27	Keratin, type I cytoskeletal 27	-61.7252	8.79879	4	17	459	6.535948068
F5H5D3	TUBA1C	Tubulin alpha-1C chain	-35.8626	8.09999	4	8	519	14.06551003
P06576	ATP5B	ATP synthase subunit beta, mitochondrial	-76.8752	9.04948	3	14	529	10.01890376
A0A024R971	FMOD	Fibromodulin, isoform CRA_a	-62.2183	7.95806	3	13	376	11.17021292
Q96HG5	ACTB	Actin, cytoplasmic 1	-29.6142	8.05816	3	8	375	13.86666596
A0A024R3X4	HSPD1	Heat shock 60kDa protein 1 (Chaperonin), isoform CRA_a	-46.8671	7.89707	2	10	573	6.282722205
Q9UDE8	LDHA	L-lactate dehydrogenase A chain	-21.0882	8.33109	2	4	332	11.14457846
Q9NNW2	CD46	Membrane cofactor protein	-15.7706	7.9736	2	4	392	5.867347121
P04745	AMY1A	Alpha-amylase 1	-14.1367	7.32082	2	4	511	5.283757299
Q96HX7	HSP90AA1	HSP90AA1 protein	-13.8499	7.57854	2	4	422	11.37440726
Q14532	KRT32	Keratin, type I cuticular Ha2	-12.0796	7.81125	2	4	448	5.133928731
Q96K68		cDNA FLJ14473 fis, clone MAMMA1001080, highly similar to Homo sapiens SNC73 protein (SNC73) mRNA	-11.5567	8.02583	2	4	494	12.55060732
A0A024R6G3	FBLN5	Fibulin 5, isoform CRA_b	-7.29757	7.87236	2	2	448	6.473214179

Removed: samples with <2 peptides, length<200, and length>600.

Supplementary Figure 1. (a) The serum from mice immunized with HEK293T whole cells or HEK293T cell-derived membrane fraction (50µg) were subjected to flow cytometry using HEK293T cells. An anti-mouse IgG conjugated with Alexa^{647} was utilized for detecting bound antibody by flow cytometry. (b) Binding characteristics of the 260 clones of the monoclonal antibody library that bind to ARPE-91 cells by flow cytometry analysis. (c) Amino acid sequence of the CDR heavy and light chain of mAb 2E7/12H8. (d) Mass spectroscopy data from 60kDa and 50kDa polypeptides excised from the 2E7 immunoprecipitation. CD46 is highlighted in red. The GEL Electrophoresis Liquid Chromatography-Tandem Mass Spectrometry (GeLC-MS/MS) analysis was performed by Bioproximity, Inc.

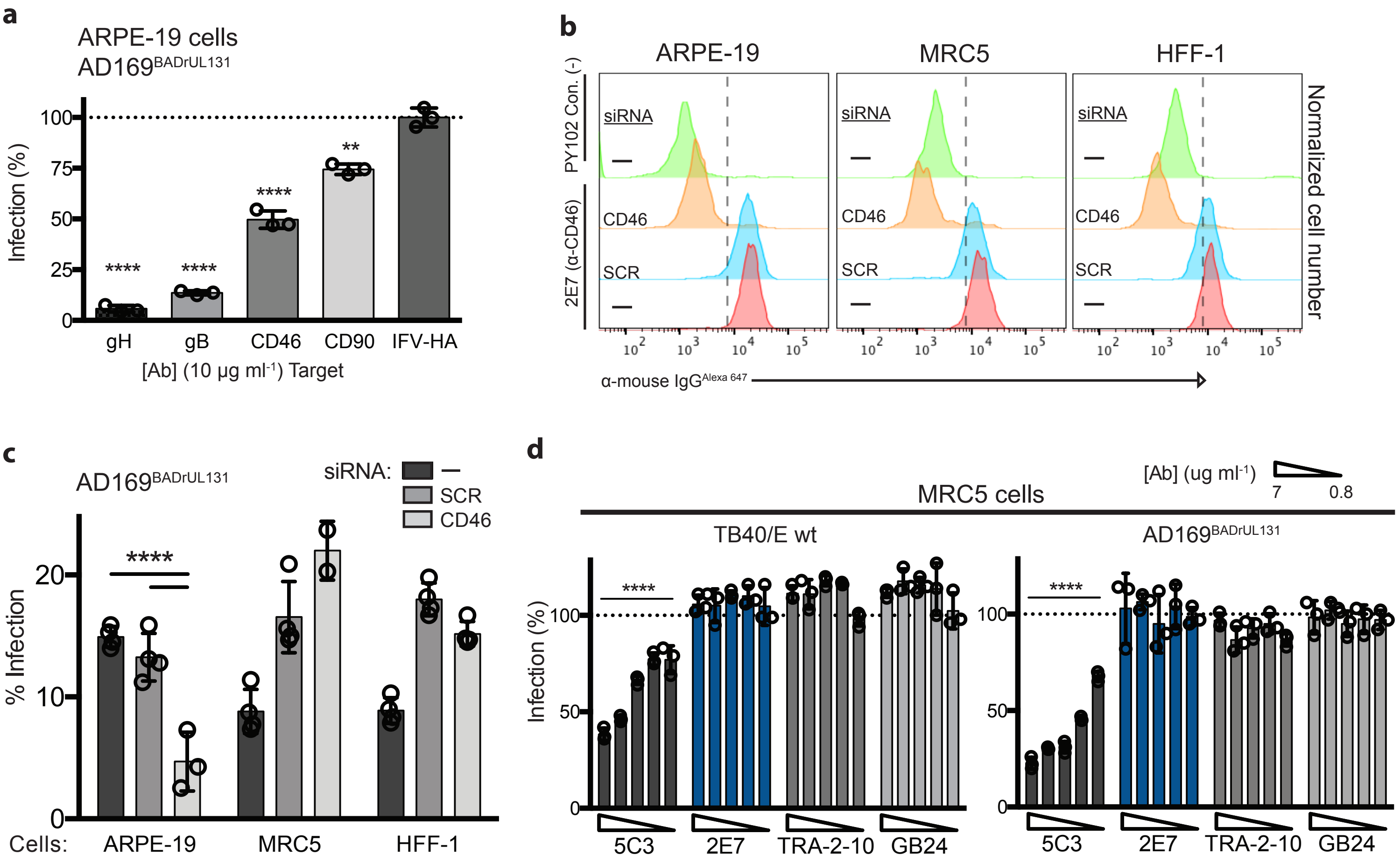

Supplementary Figure 2. (a) AD169^{BADrUL131} infected (MOI:5) ARPE-19 was subjected to a HTI with mAbs against gH (5C3), gB (ITC88), CD46 (2E7), CD90 (5E10), or influenza virus-hemagglutinin (IFV-HA)(PY102) at 10µg ml^{-1}. (b) Cell surface expression of CD46 was analyzed by flow cytometry in ARPE-19, MRC5 and HFF-1 cells transfected with no siRNA, non-targeting siRNA (SCR), or anti-CD46 siRNA. (c) ARPE-19, MRC5, and HFF-1 cells transfected with no siRNA, SCR, or anti-CD46 siRNA were infected with AD169^{BADrUL131} (MOI:0.5) and analyzed for virus infection using a Celigo Cytometer. The % of virus-infected cells was determined based on total number of cells/well. (d) TB40/E wt and AD169^{BADrUL131} (MOI:0.5) infected MRC5 cells were treated with mAb 5C3, or anti-CD46 mAbs 2E7, TRA-2-10, and GB24 and analyzed using a HTI (6.7-0.74µg ml^{-1}, in 3-fold dilutions). The % infection was determined using PY102-treated cells as 100% infection. Infection experiments were performed in triplicate. s.d. is depicted in the experiment. **P<0.01, ****P<0.0001 (Student's two-tailed *t* test).

a

```
CD46 gRNA:                                                 GCAAATGGACTTACGA GTT
Exon 3 wt: GAAACATGTCCATATATACGGGATCCTTTAAATGGCCAAGCAGTCCCTGCAAATGGACTTACGA GTTTGGTTATCAGATGCACTTTATTTGTAATGAGGG
      EC1: GAAACATGTCCATATATACGGGATCCTTTAAATGGCCAAGCAGTCCCTGCAAATGGGACTTAC-- --TTGGTTATCAGATGCACTTTATTTGTAATGAGGG
      EC2: GAAACATGTCCATATATACGGGATCCTTTAAATGGCCAAGCAGTCCCTGCAAATGGGACTTACGAAGTTTGGTTATCAGATGCACTTTATTTGTAATGAGGG
      EC3: GAAACATGTCCATATATACGGGATCCTTTAAATGGCCAAGCAGTCCCTGCAAATG---------- -TTTGGTTATCAGATGCACTTTATTTGTAATGAGGG
      TC1: GAAACATGTCCATATATACGGGATCCTTTAAATGGCCAAGCAGTCCCTGCAAATGGGACTTACGAAGTTTGGTTATCAGATGCACTTTATTTGTAATGAGGG
      TC2: GAAACATGTCCATATATACGGGATCCTTTAAATGGCCAAGCAGTCCCTGCAAAT----CT---GA -TTTGGTTATCAGATGCACTTTATTTGTAATGAGGG

 β2m gRNA: GGCCGAGATGTCTCGCT CCG
Exon 1 wt: GGCCGAGATGTCTCGCT CCGTGGCCTTAGCTGTGCTCGCGCTACTCTCTCTTTCTGGCCTGGAGGGCATCCAGC
      Eβ1: GGCCGAGATGTCTCGCTTCCGTGGCCTTAGCTGTGCTCGCGCTACTCTCTCTTTCTGGCCTGGAGGGCATCCAGC
      Tβ1: GGCCGAGATGTCTCGCTTCCGTGGCCTTAGCTGTGCTCGCGCTACTCTCTCTTTCTGGCCTGGAGGGCATCCAGC

  Insertion: X
   Deletion: -
Splice site: X
```

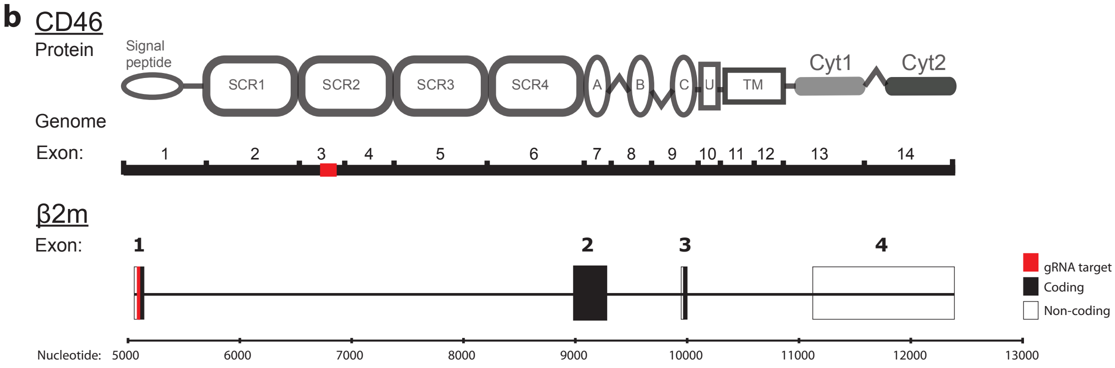

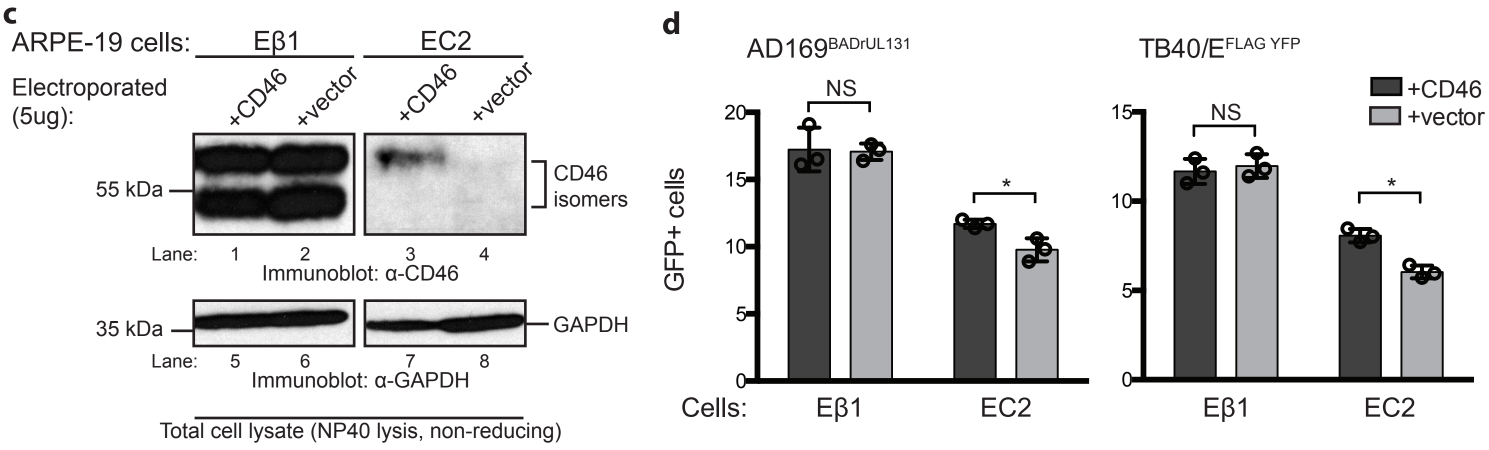

e

CMV strain: (Ewt at 100%)		TB40/E wt	SD (%)	TB40/E$^{MC\ UL99\text{-}eGFP}$	SD (%)	AD169^{BADrUL131}	SD (%)
Cell	Ewt	100.0%	0.6	100.0%	19.9	100.0%	4.5
	Eβ3	83.4%	16.3	67.2%	17.3	85.4%	7.9
	EC1	32.4%	9.6	13.1%	12.4	19.0%	3.4
	EC2	28.7%	4.6	16.4%	2.8	35.0%	2.2
	EC3	27.6%	8.3	19.7%	9.9	25.5%	7.7

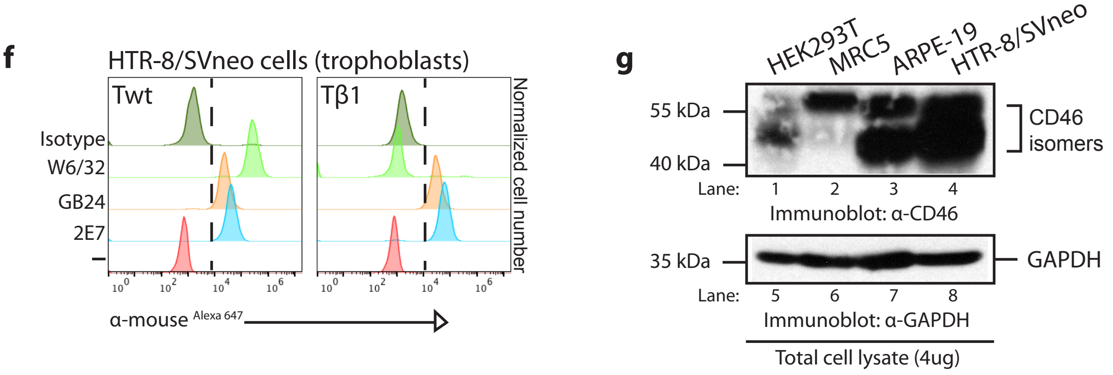

Supplementary Figure 3. (a) CD46 and β_2-microglobulin (β_2m) gRNAs were aligned to the corresponding sequences of *CD46* and $\beta_2 m$ cloned from knock-out ARPE-19 (E) and HTR-8/SVneo (T) clones. Insertions are indicated in red, deletions with a dash, and putative edited splice site (predicted with http://www.fruitfly.org/seq_tools/splice.html[37]) in green. (b) gRNAs designed to target *CD46* and $\beta_2 m$. (c) CD46 or a vector control were electroporated into Eβ1 and EC2 cells and lysates were subjected to immunoblot analysis for CD46 (lanes 1-4) and GAPDH (lanes 5-8). (d) Electroporated Eβ1 and EC2 cells were infected with AD169^{BADrUL131} and TB40/E wt and GFP+ cells were determined by flow cytometry. The total percentage of infected cells was determined and compared to the electroporation/infection conditions. (e) Quantification of plaque reduction (%) in ARPE-19 clones from Fig. 6e. (f) β_2m knock-out was analyzed through the evaluation of MHC class I (HLA-C and -G in trophoblasts) cell surface expression in HTR-8/SVneo β_2m-KO (Tβ1) by flow cytometry. Binding of mAb 2E7 and GB24 confirms structural integrity of CD46, mAb W6/32 confirms correct folding of MHC class I, and PY102 is a non-binding control. (g) HEK293T, MRC5, ARPE-19, and HTR-8/SVneo cell lysates were subjected to immunoblot analysis for CD46 (lanes 1-4) and GAPDH (lanes 5-8). Infection experiments were performed in triplicate. s.d. is depicted in the experiment. $^*P<0.05$ (Student's two-tailed *t* test).

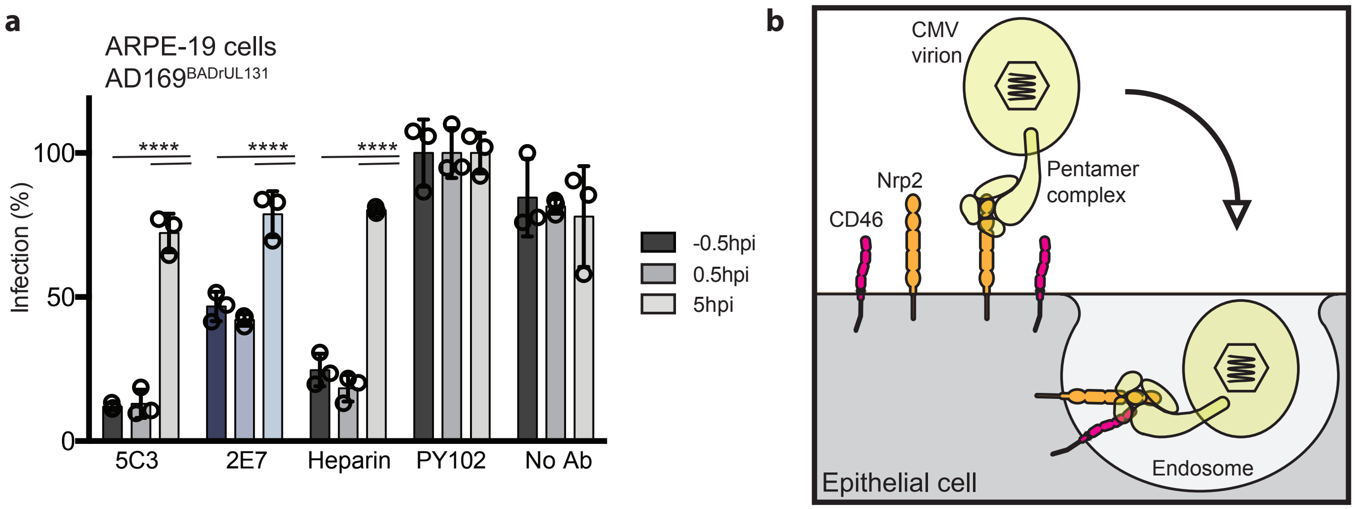

Supplementary Figure 4. (a) AD169 ^{BADrUL131} was added to ARPE-19 cells untreated or treated at -0.5hpi, 0.5hpi, and 5hpi with mAbs 5C3, 2E7, and PY102, heparin, or a no-antibody control (10μg ml^{-1}). (b) Schematic depiction of the temporal interaction of Nrp2 and CD46 with CMV during virus entry into epithelial cells. Infection experiments were performed in triplicate. s.d. is depicted in the experiment. ****P<0.0001 (Student's two-tailed *t* test).
