# Supplementary material for: CD46 facilitates entry and dissemination of human cytomegalovirus
Source: Nat Commun. 2019 Jun 20;10:2699. doi: 10.1038/s41467-019-10587-1 (PMC6586906; doi:10.1038/s41467-019-10587-1)
Supplement: Supplementary file 2 — Reporting Summary [file 41467_2019_10587_MOESM2_ESM.pdf]

nature research

Corresponding author(s): Domenico Tortorella

Last updated by author(s): May 3, 2019

Reporting Summary

Nature Research wishes to improve the reproducibility of the work that we publish. This form provides structure for consistency and transparency in reporting. For further information on Nature Research policies, see Authors & Referees and the Editorial Policy Checklist.

Statistics

For all statistical analyses, confirm that the following items are present in the figure legend, table legend, main text, or Methods section.

n/a	Confirmed	
☐	☒	The exact sample size (*n*) for each experimental group/condition, given as a discrete number and unit of measurement
☐	☒	A statement on whether measurements were taken from distinct samples or whether the same sample was measured repeatedly
☐	☒	The statistical test(s) used AND whether they are one- or two-sided<br>*Only common tests should be described solely by name; describe more complex techniques in the Methods section.*
☒	☐	A description of all covariates tested
☒	☐	A description of any assumptions or corrections, such as tests of normality and adjustment for multiple comparisons
☐	☒	A full description of the statistical parameters including central tendency (e.g. means) or other basic estimates (e.g. regression coefficient) AND variation (e.g. standard deviation) or associated estimates of uncertainty (e.g. confidence intervals)
☐	☒	For null hypothesis testing, the test statistic (e.g. *F*, *t*, *r*) with confidence intervals, effect sizes, degrees of freedom and *P* value noted<br>*Give P values as exact values whenever suitable.*
☒	☐	For Bayesian analysis, information on the choice of priors and Markov chain Monte Carlo settings
☒	☐	For hierarchical and complex designs, identification of the appropriate level for tests and full reporting of outcomes
☒	☐	Estimates of effect sizes (e.g. Cohen's *d*, Pearson's *r*), indicating how they were calculated

Our web collection on statistics for biologists contains articles on many of the points above.

Software and code

Policy information about availability of computer code

Data collection: *Provide a description of all commercial, open source and custom code used to collect the data in this study, specifying the version used OR state that no software was used.*

Data analysis: *Provide a description of all commercial, open source and custom code used to analyse the data in this study, specifying the version used OR state that no software was used.*

For manuscripts utilizing custom algorithms or software that are central to the research but not yet described in published literature, software must be made available to editors/reviewers. We strongly encourage code deposition in a community repository (e.g. GitHub). See the Nature Research guidelines for submitting code & software for further information.

Data

Policy information about availability of data

All manuscripts must include a data availability statement. This statement should provide the following information, where applicable:

- Accession codes, unique identifiers, or web links for publicly available datasets
- A list of figures that have associated raw data
- A description of any restrictions on data availability

All data is provided in the Source Data file.

Field-specific reporting

Please select the one below that is the best fit for your research. If you are not sure, read the appropriate sections before making your selection.

☒ Life sciences ☐ Behavioural & social sciences ☐ Ecological, evolutionary & environmental sciences

Life sciences study design

All studies must disclose on these points even when the disclosure is negative.

Sample size	All experiments were performed in triplicate or otherwise noted. All data points were included in the figures.
Data exclusions	No data exclusions.
Replication	All have been replicated at least twice.
Randomization	Not applicable.
Blinding	Not applicable.

Reporting for specific materials, systems and methods

We require information from authors about some types of materials, experimental systems and methods used in many studies. Here, indicate whether each material, system or method listed is relevant to your study. If you are not sure if a list item applies to your research, read the appropriate section before selecting a response.

Materials & experimental systems

n/a	Involved in the study	
☐	☒	Antibodies
☐	☒	Eukaryotic cell lines
☒	☐	Palaeontology
☐	☒	Animals and other organisms
☒	☐	Human research participants
☒	☐	Clinical data

Methods

n/a	Involved in the study	
☒	☐	ChIP-seq
☐	☒	Flow cytometry
☒	☐	MRI-based neuroimaging

Antibodies

Antibodies used	Anti-CD46 immunoglobulins were raised in rabbits by inoculation with aa36-140 of CD46 (GenScript, Piscataway, NJ). The human cytomegalovirus (CMV) anti-immediate early-1 (IE1) immunoglobulins were raised in rabbit by inoculation with a peptide derived from the CMV TB40/E sequence shared by the IE1 and IE2 protein38 (GenScript). The CMV neutralizing gH mAb 5C3 was purified from hybridoma culture supernatant29. The mAb PY102, utilized as a non-binding control, recognizes the globular head of the HA of PR/8 (H1N1) influenza A virus.39 The mAb W6/32, which recognizes properly folded MHC class I molecules, was purified from hybridoma culture supernatant. Anti-CD46 mAbs TRA-2-1025 and GB2426 were gifts from Dr. J P Atkinson (Washington University, St. Louis, MO). Anti-CD90 mAb 5E10 (Thermo Fisher), anti-glyceraldehyde-3-phosphate dehydrogenase (GAPDH) (Chemicon) and anti-Nrp2 (Thermo Fisher-257103) were purchased. The mAbs 2E7, 12H8, and anti-gB ITC88 production are described in methods.
Validation	The primary antibodies were validated through various experimental strategies including western blots, flow cytometry, and immunoprecipitation studies.

Eukaryotic cell lines

Policy information about cell lines

Cell line source(s)	Human retinal epithelial ARPE-19 cells (ATCC #CRL-2302) were cultured in a 1:1 ratio of Dulbecco's modified Eagle's medium (DMEM) to Kaighn's modification of Ham's F-12 medium (F-12k). A549 human lung epithelial cells (ATCC #CCL-185) were cultured in F-12k. Jurkat, Clone E6-1 (ATCC #TIB-152) peripheral blood T lymphocytes and HTR-8/SVneo (ATCC #CRL-3271) were cultured in RPMI-1640 medium. MRC5 lung fibroblasts (ATCC #CCL-171), HFF-1 (ATCC SCRC-1041), and HEK293T (ATCC #CRL-3216) were cultured in DMEM.
Authentication	All cell lines were commercially available and validated by the supplier.
Mycoplasma contamination	We confirm that the cell lines tested negative for mycoplasma.
Commonly misidentified lines (See ICLAC register)	None

Animals and other organisms

Policy information about studies involving animals; ARRIVE guidelines recommended for reporting animal research

Laboratory animals	Female BALB-C mice
Wild animals	Not applicable
Field-collected samples	Not applicable
Ethics oversight	Icahn School of Medicine at Mount Sinai Institutional Animal Care and Use Committees (IACUC)

Note that full information on the approval of the study protocol must also be provided in the manuscript.

Flow Cytometry

Plots

Confirm that:

☒ The axis labels state the marker and fluorochrome used (e.g. CD4-FITC).

☒ The axis scales are clearly visible. Include numbers along axes only for bottom left plot of group (a 'group' is an analysis of identical markers).

☐ All plots are contour plots with outliers or pseudocolor plots.

☒ A numerical value for number of cells or percentage (with statistics) is provided.

Methodology

Sample preparation	The respective cells were blocked with 1%BSA/PBS (4°C), incubated with primary antibody (2μg ml-1) or hybridoma supernatant at specified dilutions followed by goat anti-mouse conjugated with Alexa647 (4μg ml-1)(ThermoFisher Scientific) and analyzed using an Intellicyt HTFC flow cytometer (Intellicyt). For analysis of permeabilized cells, cells were fixed in BD Biosciences Cytofix/Cytoperm solution (20 min 4° C) and maintained in 0.1% saponin.
Instrument	Intellicyt HTFC flow cytometer
Software	Flow Jo software (Tree Star, Inc)
Cell population abundance	Not applicable.
Gating strategy	Not applicable.

☐ Tick this box to confirm that a figure exemplifying the gating strategy is provided in the Supplementary Information.
